# Building consensus on core teaching content of a digital public health curriculum: a Delphi study with public health experts in Germany

**DOI:** 10.3389/fpubh.2026.1799393

**Published:** 2026-06-19

**Authors:** Laura Maaß, Anna Lea Stark-Blomeier, Pinar Tokgöz, Florian Fischer, Henriette Schulz, Joanna Albrecht

**Affiliations:** 1 SOCIUM Research Center on Inequality and Social Policy, University of Bremen, Bremen, Germany; 2 Leibniz ScienceCampus Digital Public Health Bremen, Bremen, Germany; 3 German Public Health Association (DGPH), Digital Public Health Section, Berlin, Germany; 4 Chair of Digital Public Health, Department of Social Sciences, Faculty of Arts and Humanities, University of Siegen, Siegen, Germany; 5 Institute for Public Health, Charité – Universitätsmedizin Berlin, Berlin, Germany; 6 Bavarian Research Center for Digital Health and Social Care, Kempten University of Applied Sciences, Kempten, Germany; 7 Faculty III: Health, Medical and Life Sciences, Furtwangen University, Furtwangen, Germany

**Keywords:** competencies, education, Germany, public health, training, university

## Abstract

**Introduction:**

The digital transformation has substantially altered the competencies required of public health professionals. In Germany, digital public health (DiPH) content is inconsistently integrated into public health degree programs, reflecting the absence of a unified educational framework. To address this gap, this study aimed to establish expert consensus on core teaching content for a DiPH curriculum tailored to public health education in Germany.

**Methodology:**

A multi-stage Delphi study was conducted between May 2024 and May 2025, involving public health experts from various public health settings to address scientific and practice-related expertise. Three online Delphi rounds were conducted. Experts rated the relevance of predefined and participant-generated teaching content on a 9-point Likert scale. Consensus was defined *a priori* as a mean score ≥7.0. Teaching content was iteratively included, excluded, or re-evaluated across rounds and clustered into eleven predefined public health topic areas. In the final round, experts additionally prioritized teaching content within topic areas and identified the ten most important content areas overall.

**Results:**

Across the three Delphi rounds, 129 teaching contents were assessed. Consensus was achieved on 80 core teaching content items, spanning 11 public health topic areas. Most content reached consensus after their first presentation. The largest number of teaching content was identified in the domain “Methods in the social sciences,” followed by “Health communication” and “Health promotion, education, and prevention.” Prioritization exercises highlighted participatory and user-centered approaches, the development and evaluation of DiPH interventions, foundational concepts of digital health and DiPH, and ethical and legal requirements for digital innovations as among the most important teaching content. The final top ten teaching content represented nine of the eleven topic areas.

**Conclusion:**

This study provides a comprehensive, consensus-based set of core teaching content for DiPH education within public health degree programs that can be applied beyond the German context. The findings reflect the breadth and interdisciplinarity of public health in the digital age and emphasize competencies related to co-creation, evaluation of digital interventions, digital health literacy, ethics, and health system transformation. The resulting core curriculum provides a structured foundation for harmonizing DiPH education and preparing the next public health workforce.

## 1 Introduction

For more than three decades, public health degree programs in Germany have evolved (Blättner & Dierks, 2021) with a focus on preventing disease, promoting health, and prolonging life, while accounting for the equitable distribution and efficient use of available resources (1). Relevant degree programs should ensure the production of qualified, interdisciplinary, and practice-oriented professionals (2). This principle of public health qualification still applies today, even though the structure of the training landscape has changed significantly.

With the digital transformation of health (including healthcare services, health promotion, or population surveillance), new demands arise for public health graduates (3). Though the advantages of digital technologies are numerous, they can only realize their full potential if the next generation of public health professionals has knowledge and competencies in this area and can interact with these technologies (4). It is therefore of great importance that public health degree programs reflect these changes and ensure that graduates have the necessary expertise and skills to meet future challenges in the digital healthcare landscape (5).

In this context, digital public health (DiPH) as a means to an end for realizing public health goals gains significance (6–8). For this reason, a study of current public health degree programs focused on distinct modules taught at DiPH at public German universities. Among the 16 identified programs, a variety of content related to DiPH was found. Modules ranged from ethical and legal aspects to the digitalization of healthcare, DiPH regulation and governance, health promotion applications, and questions regarding equity and the social and digital divide (9).

These findings showcase the diversity of DiPH and highlight the lack of a standardized DiPH education within public heath programs in Germany. Consequently, the need for a unified definition of core teaching content in DiPH and of content to be integrated into public health education is obvious. While the core curriculum for public health of the Association of Schools of Public Health in the European Region (ASPHER) lists essential competencies, including DiPH (10), the relevance of these competencies for public health education in Germany remains unclear. It is important to note that public health in Germany is taught as independent Bachelor's and Master's degree programs, rather than as a specialization within a medical degree program with a subsequent Master's degree in public health, as in other countries such as Italy or the United Kingdom. Primary clinical content and the digitalization of medicine are, therefore, only of secondary relevance in public health training in Germany. However, they frequently occur in international competence frameworks for the public health workforce, such as the ASPHER framework (10).

Against this background, we conducted a multi-stage Delphi study with public health experts from academia, governance, and practice to aim for consensus on DiPH teaching content most essential for the public health education in Germany. As such, our study aims to identify and prioritize core teaching content for DiPH, thereby creating a curriculum framework for DiPH education, that is, a structured set of core content domains intended to guide future DiPH curriculum development. To ensure conceptual grounding, our approach is informed by the ASPHER core competencies framework and our systematic analysis of module handbooks from DiPH programs in Germany (9, 10). The following perspectives guided the Delphi study in reaching a consensus:

1. DiPH is as diverse and heterogeneous in terms of its topics as public health.
2. A core curriculum in DiPH reflects this diversity; consequently, it is not (exclusively) limited to one sub-area of public health.

## 2 Materials and methods

The study was conducted by public health experts with a background in DiPH, extensive experience in developing and monitoring DiPH programs, and who had previously conducted a study on DiPH-related modules currently taught within public health programs in Germany (9).

### 2.1 Study design

The Delphi method is a well-established social science approach for achieving expert consensus on complex topics, such as DiPH (11, 12). In our study, experts were invited to independently express their opinions on a core curriculum in a multi-stage survey process with several rounds. The individual responses from each round were pooled, analyzed using qualitative content analysis, and then presented to participants again to initiate consensus-building (13). In this way, consensus among the experts on essential teaching contents was achieved without favoring or disadvantaging any individual participant (11). We used an online pre-survey to register interested experts, followed by three online rounds using the General Data Protection Regulation (GDPR)-compliant platform LimeSurvey.

### 2.2 Expert selection

Our Delphi study included experts from various public health-related settings and thus addressed scientific and practice-related experts. The pre-defined inclusion and exclusion criteria are displayed in Table 1. Based on our previous experience with Delphi procedures (7), we estimated a 50% dropout rate among experts throughout our study. Consequently, we decided to start the first Delphi round after 40 experts had registered via the preliminary survey (R0). This was anticipated to yield a sufficient cohort of 7 to 15 experts in the third Delphi round (R3), as suggested by Fitch et al. (14). Following other Delphi studies aimed at reducing attrition bias, we invited only experts who had participated in the previous survey round to the follow-up panel (15).

**Table 1:** Inclusion and exclusion criteria for the Delphi study.

Inclusion criteria	Exclusion criteria
-18 years of age or older -written electronic consent to participate -experience in public health teaching, research, or practice for at least 1 year -at least 1 year of experience with the digitalization of health -current professional activity (employment) in Germany in at least one of the following settings with relation to the digitalization of health and health systems: -lecturer or researcher in a public health program at a university -health insurance company -national or regional Ministry of Health -public health service -health technology company -nanother public health institution	-people unable to complete the online questionnaire (e.g., due to a lack of time or internet availability) -lack of language comprehension (German) - incomplete fulfillment of the inclusion criteria

**Figure 1:**
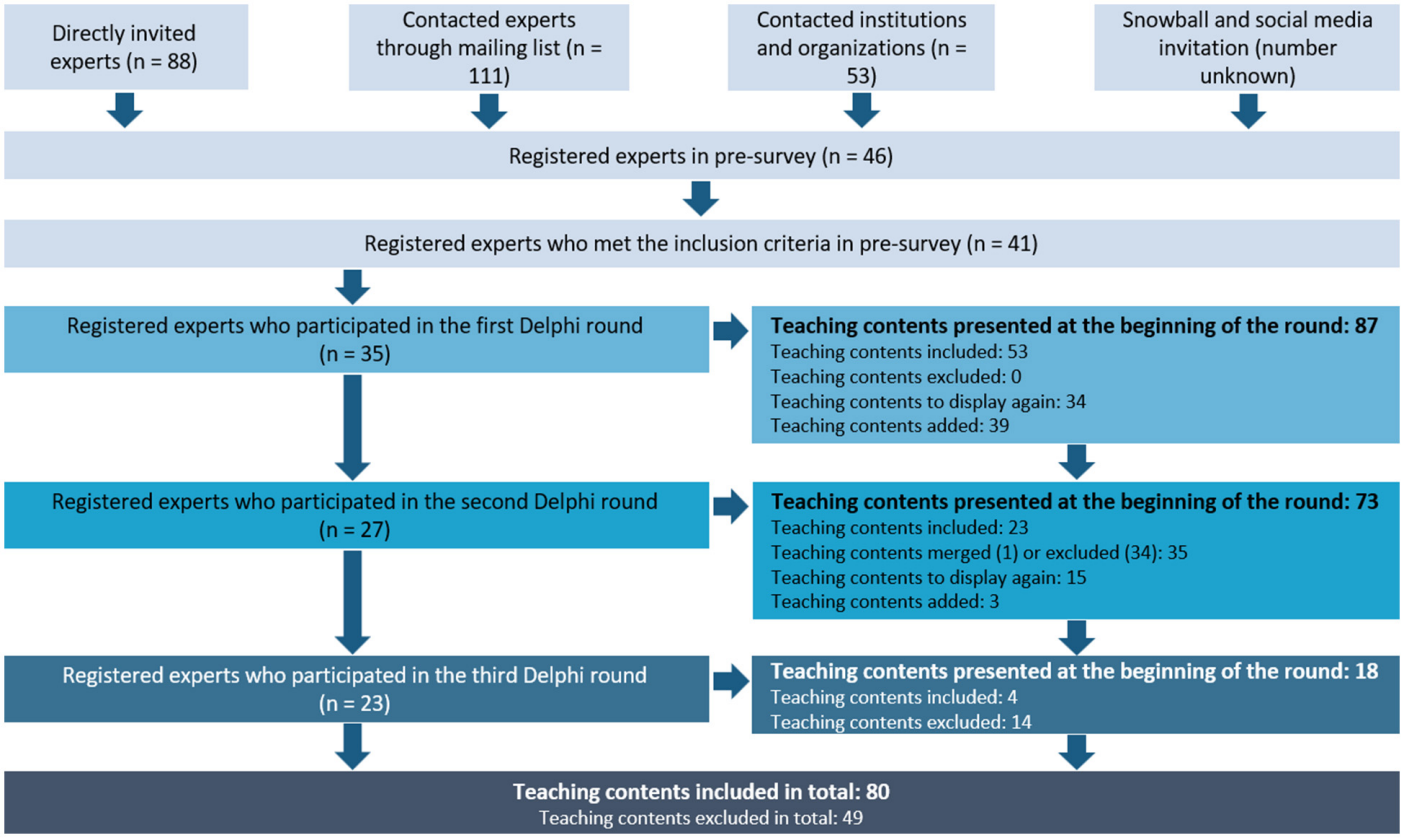
Flow chart of participants and the number of teaching contents displayed per round.

Experts were recruited via direct email contact (contact details were obtained from their institutional websites or from their published articles as corresponding authors), through the newsletter of the German Public Health Association (DGPH) Digital Public Health section, and on LinkedIn. Further, we applied a snowballing method to increase the reach of interested experts (16) and reached out to (research) institutions, health insurances, non-government organizations, the national public health agency, and the regional and national ministries of health and politicians involved in the digitalization of healthcare in Germany. The total number of contacted individuals and institutions is displayed in Figure 1.

### 2.3 Questionnaire design and study procedure

This Delphi study followed a pseudonymized approach where the participants chose an alias in R0 to later log into the official Delphi rounds R1–R3. The entire study was conducted on the open-source platform LimeSurvey. Pseudonymized approaches in qualitative studies (particularly in Delphi studies) have been shown to encourage freer expression of ideas and reduce the risk of dominant voices (17). The questionnaires were pre-tested with members of the Digital Public Health section of the DGPH who did not participate in the study. For this purpose, the respondents were asked to imagine themselves as program directors designing a DiPH curriculum for their public health degree program, with the option to choose predefined course content and add others to create their ideal curriculum. The feedback was provided in written form.

Before recruitment, we qualitatively assessed and clustered the teaching content identified in our previous analysis of DiPH programs in Germany and the ASPHER core competencies framework (9, 10) using the thematic analysis approach by Braun and Clarke (18). This resulted in 80 teaching topics that were grouped across the following public health settings as topic areas:

1. Fields of application of digital interventions in healthcare.
2. Health policy and systems.
3. Methods in the social sciences.
4. IT and technology.
5. Ethics and law
6. Epidemiology.
7. Health communication.
8. Health promotion, education, and prevention.
9. Health economics and management.
10. Public health and social medicine.
11. Determinants of health and illness and social inequalities.

During the Delphi study, we analyzed participants' proposed teaching content using the same methodological approach and clustered it into the aforementioned 11 overarching topic areas.

In R0 (from May to October 2024), all participants were electronically provided with information on data protection and processing for this study. Those who provided their informed electronic consent proceeded to the second block, were asked for contact details, and set an alias to later connect their responses from the upcoming Delphi rounds (pseudonym). Finally, we collected sociodemographic data (including the highest level of education, work-related setting, primary subject area, years of experience, nationality, gender, and year of birth).

During R1-R3 (from October 2024 to May 2025, each lasting 5 weeks), experts were provided with definitions of public health and DiPH based on Gerlinger et al. (1) and Odone et al. (8). According to them, public health focuses on preventing disease, prolonging life, and promoting health on a population level, whereas DiPH is the digitization of methods and applications to achieve traditional public health goals (1, 8). We further informed participants that additional suggestions should be phrased as teaching content for public health education, in line with the Organization for Economic Co-operation and Development's (19) definition of competencies. Hereby, a competency includes knowledge, skills, attitudes, and the capability to meet complex requirements in a particular setting (19). Therefore, we defined teaching content as the topics, concepts, or learning objectives to be taught in public health education, enabling students to develop DiPH-related competencies.

For R2 and R3, they were also shown the overall average vote of each teaching content displayed in the previous round. They then rated the relevance of the presented teaching content on a 9-point Likert scale (1 = “not important at all,” 9 = “very important”). In addition to voting on the importance of teaching content in R1 and R2, experts were invited to suggest additional content or rephrase the provided content. Additionally, participants could select “I cannot rate this indicator due to a lack of expertise” if they felt that the respective teaching content was beyond their expertise. Across all rounds, experts were invited to the upcoming Delphi questionnaire only if they had participated in the previous survey, to reduce the risk of attrition bias. However, this approach resulted in a decreasing panel size throughout the study (see Table 2).

**Table 2:** Characteristics of the study participants between the rounds.

Item		Round 1 *n* (%) (*n* = 35)	Round 2 *n* (%) (*n* = 27)	Round 3 *n* (%) (*n* = 23)
Age (mean in years)		43	44	45
Gender, *n* (%)	Female	19 (54.28)	14 (51.85)	12 (52.17)
	Male	15 (42.86)	13 (48.15)	11 (47.83)
	Diverse	1 (2.86)	–	–
Nationality, *n* (%)	German	34 (97.14)	27 (100.00)	23 (100.00)
	Mexican	1 (2.86)	–	–
Degree, *n* (%)	Bachelor	2 (5.71)	1 (3.70)	1 (4.35)
	Master	13 (37.14)	9 (33.34)	7 (30.43)
	Diploma	1 (2.86)	1 (3.70)	1 (4.35)
	PhD	18 (51.43)	15 (55.56)	13 (56.52)
	Habilitation	1 (2.86)	1 (3.70)	1 (4.35)
Occupation, *n* (%)	Teacher/Researcher in public health	19 (54.27)	16 (59.26)	16 (69.57)
	Teacher/Researcher outside of public health	4 (11.45)	3 (11.11)	3 (13.04)
	Public health service	3 (8.57)	3 (11.11)	1 (4.35)
	Health technology company	3 (8.57)	2 (7.41)	–
	Public health institution	3 (8.57)	1 (3.70)	1 (4.35)
	Ministry of Health	2 (5.71)	2 (7.41)	2 (8.69)
	Health insurance	1 (2.86)	–	–
Main field of work, *n* (%)	Public health	19 (54.27)	15 (55.56)	13 (56.51)
	Medicine	3 (8.57)	2 (7.42)	2 (8.69)
	Epidemiology	3 (8.57)	2 (7.42)	–
	Economic sciences	2 (5.71)	1 (3.70)	1 (4.35)
	Other	8 (22.88)	7 (25.90)	7 (30.45)
Professional experience (mean in years)	Main field of work	12	12	13
	Public health	10	11	10
	Digital public health	6	6	6
	Digital health	7	8	8

In the planned fourth Delphi round, the agreed teaching content was to be sorted by its importance for the individual subject areas and overall. However, after R2, only a few teaching contents failed to achieve consensus or were newly proposed, so we decided to combine R4 with R3 to limit the risk of losing too many participants and conduct a meaningful sorting of teaching content. Consequently, experts were asked to rank included teaching contents from R1 and R2, as well as the remaining contents from R3, for each public health area and for the overall top 10 based on their importance for a DiPH curriculum. While participants ranked every teaching content within each public health domain based on its relevance to a DiPH curriculum, a pre-test showed that the same approach would be too time-consuming for identifying the top 10 teaching contents overall. Therefore, participants were asked to select their top 10 most important teaching contents for contemporary public health education, without ranking them by importance. Teaching content that did not reach consensus on inclusion in R3 was retrospectively removed from the ranking exercise.

Consensus was defined *a priori* as follows: We included teaching content with an average Likert score among all participating expert groups of at least 7.0. Content with a score below 4.0 was excluded. If a teaching content received an average vote between both cut-off values, it was presented once more. If consensus remained absent after a second presentation, the teaching content was deemed excluded.

### 2.4 Data analysis

The descriptive analysis of responses and sub-analysis were conducted with Microsoft Excel 2019. One author (LM) extracted the pseudonymized responses from LimeSurvey, which were then analyzed by three other authors independently (ALSB, PT, and JA). Since we combined R3 and R4, we conducted a sensitivity analysis of the remaining 18 teaching contents that lacked consensus in R3. This was necessary because we included all 18 remaining teaching contents in the ranking from most to least important per topic area, and the overall selection of the top 10 teaching contents. However, before R3, it was unclear which of the 18 teaching contents the participants would agree on. The aim of this analysis was, therefore, to identify differences in the ranking when all 18 teaching contents from R3 were considered, compared to when only those agreed upon during R3 were considered. The result of this analysis is displayed in Supplementary material 2.

Furthermore, we conducted a content analysis of the discussed teaching content (both those with consensus and those excluded) to compare the relevance of our findings with the ASPHER core-competence framework's section on digital transformation in public health (10) and the Global Competence and Outcome Framework of the World Health Organization (WHO) (20).

### 2.5 Ethical approval

Ethical approval was obtained from the University of Siegen (approval number: LS_ER_17_2024). Informed consent was obtained from each participant at the beginning of R0, prior to the collection of sociodemographic data and contact details. Participants were assured of the confidentiality and pseudonymity of this study, as well as their right to withdraw at any time.

## 3 Results

### 3.1 Expert sociodemographic information

A total of 46 experts participated in R0 (registration), of which 41 met the inclusion criteria and were invited to R1. Of these, 35 participated in R1 (85.37% response rate). For R2, only 27 of the 35 experts (77.14% response rate) completed the survey, and 23 of the invited 27 experts (85.19% response rate) participated in R3. This results in an overall response rate of 50%. In R1 and R2, all participating experts completed the survey. However, in R3, three experts dropped out after ranking the teaching contents on Likert scales (thereby not contributing to the identification of the 10 most important teaching contents).

Participants' demographics and professional background are presented in Table 2. The gender distribution was almost equal, with 52%-54% of female experts in each Delphi round. Apart from R1, all participants stated that they were German nationals. In R1, one person was Mexican. Over the three rounds, the participants' average age ranged from 43 to 45 years. Between 54% (R1) and 57% (R3) of all experts had a background in public health. Other experts came from medicine, epidemiology, and economic sciences (2–3 each per round). Other disciplines represented with one expert each were digital health, health economics, informatics, nursing sciences, psychology, and marketing (only involved in R1), as well as “interdisciplinary fields” and “general digitization” with one expert each per round. The majority of participants in R1 (51%), R2 (56%), and R3 (57%) held doctoral degrees and worked primarily as teachers and researchers in public health (54% in R1, 59% in R2, and 70% in R3).

The participants had, on average, 12 years (R1 and R2) and 13 years (R3) of experience in their primary field of work, with 10 years (R1 and R3) and 11 years (R2) of experience in public health. Their average years of experience in digital health were on average seven (R1) and 8 years (R2 and R3), and 6 years on average for DiPH.

### 3.2 Core teaching content in digital public health

#### 3.2.1 Cross-cutting inclusion and exclusion processes of the presented teaching content in R1 to R3

In the study, a total of 129 teaching contents were presented for consensus on their relevance to a DiPH curriculum (*n* = 87 literature-based teaching content and *n* = 42 participant-generated teaching content; generated in R1: *n* = 39; in R2: *n* = 3). Literature-based content included both competencies as defined in the updated ASPHER core curriculum for DiPH (10) and teaching content identified in our previous analysis of established teaching modules on DiPH in German public health programs (9). Figure 1 displays the number of teaching content items included, excluded, added, or displayed again at each of the three Delphi rounds. As such, it provides an overview of the change in items and the number of contributing experts per round. Further, Table 3 provides a more detailed overview of the development, inclusion of agreed teaching content, and exclusion in the three Delphi rounds for each of the eleven predefined public health topics.

**Table 3:** Overview of agreed teaching content over the three Delphi rounds.

Topic area	Fields of application of digital interventions in healthcare	Health policy and systems	Methods in the socialsciences	IT and technology	Ethics and law	Epidemiology	Health communication	Health promotion, education, and prevention	Health economics and management	Public health andsocial medicine	Determinants of health and illness and social inequalities
Round 1											
Shown content, *n*	15	9	11	10	7	8	8	5	3	6	5
Mean Overall, M	6.6	6.9	7.3	7	7.4	7.2	7.2	7.6	6.9	7	7.8
Excluded content, *n*	0	0	0	0	0	0	0	0	0	0	0
Uncertain content, *n*	11	4	3	6	1	3	2	0	2	2	0
Included content, *n*	4	5	8	4	6	5	6	5	1	4	5
15.6-7.4,-1.3690pt Added content, *n*	5	5	10	3	2	1	3	2	5	1	2
Round 2											
Shown content, *n*	16	9	13	9	3	4	5	2	7	3	2
Mean Overall, M	6.3	6.4	7	6.6	6.5	6.7	6.7	7.6	6.1	5.2	7.1
Excluded content, *n*	11	4	3^*^	6	1	3	2	0	2	2	0
Uncertain content, *n*	3	3	1	2	2	1	1	0	1	1	0
Included content, *n*	2	2	8	1	–	–	2	2	4	–	2
15.6-7.4,-1.3690pt Added content, *n*	1	0	1	0	0	0	0	1	0	0	0
Round 3											
Shown content, *n*	4	3	2	2	2	1	1	1	1	1	0
Mean Overall, M	6.4	6.0	7.0	6.4	6.3	6.9	7.0	7.3	6.1	5.3	–
Excluded content, *n*	3	3	1	2	2	1	0	0	1	1	0
Included content, *n*	1	0	1	0	0	0	1	1	0	0	0
**Core teaching content agreed upon**, ***n*** **(%)**	**7 (8.8)**	**7 (8.8)**	**17 (21.3)**	**5 (6.3)**	**6 (7.5)**	**5 (6.25)**	**9 (11.3)**	**8 (10.0)**	**5 (6.3)**	**4 (5.0)**	**7 (8.8)**

^*^+ 1 content merged with other teaching content.

In total, 48 teaching contents were excluded based on expert suggestions (R1: *n* = 0; R2: *n* = 34; R3: *n* = 14). Additionally, the participant-generated teaching content “Data analysis (access to data, usability, biostatistics, etc.)” was merged with the deductively presented “Identification, evaluation, and use of secondary data (e.g., Google searches, biostatistics, etc.)” at the participant's suggestion in R2. Finally, 80 core teaching contents were identified and rated by participants as “important” or “very important” (53 literature-based and 27 participant-generated). An overview of the distribution of all core teaching content by the 11 topic areas and their consensus across the three rounds is shown in Supplementary material 1. As shown in Table 3, nearly all core teaching content (*n* = 79) reached consensus immediately after their first presentation, whereas only one reached consensus after a second presentation.

The cross-cutting inclusion and exclusion process across rounds can be summarized as follows: At the outset in R1, 87 literature-based teaching contents were presented and rated on a scale from 1 to 9. No teaching content was directly excluded based on a mean score of 1.00–3.99. Instead, 53 teaching contents were agreed upon and included, while 34 were classified as uncertain in terms of importance and thus carried forward to R2. In addition, participants suggested 39 new teaching contents, which were introduced in R2. In R2, 73 teaching contents were presented for consensus (*n* = 34 uncertain contents from R1 and *n* = 39 new participant-generated contents from R1). All 34 contents carried over from R1 remained below the inclusion threshold and were excluded. 23 teaching contents were included as core teaching contents, while 15 teaching contents were classified as uncertain in terms of importance and moved forward to R3. In the final R3, 18 teaching contents were presented (*n* = 15 participant-generated from R1 and *n* = 3 new participant-generated contents from R2). Of these, 14 teaching contents (M ≤ 6.99) were ultimately excluded, while four teaching contents were included as additional core teaching content.

#### 3.2.2 Topic area-specific inclusion and exclusion processes of teaching content in R1-R3

**Figure 2:**
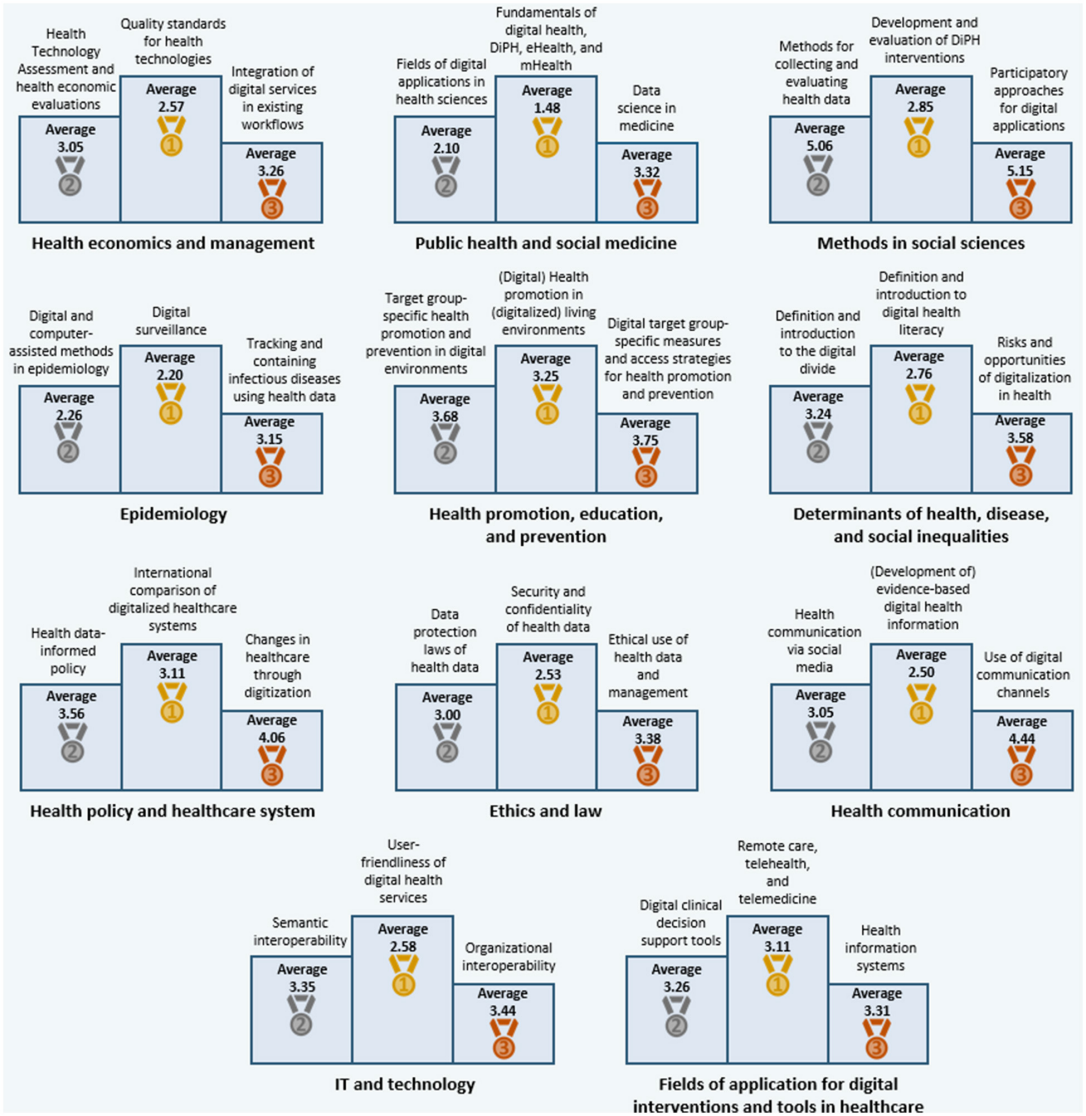
Distribution of agreed core teaching content by topic areas across the Delphi study.

An overview of the distribution of the eventually included teaching content by topic areas, and its consensus across the three rounds, is shown in Figure 2. Overall, the majority of the curriculum content (64%) was finalized during the first round of consultations. On average, 71% of the teaching content ultimately approved for each public health domain was included during R1 (ranging from 20% to 100% per domain). An overview of the concentrated core teaching content and its inclusion type is provided in Supplementary material 2.

17 of the final core teaching content are found in the topic area “Methods in the social sciences” with an average score (relevance) of 7.04 to 7.81. They can be categorized in content on data collection and processing (including the use of new sources like Social Media, wearable devices, diversity-sensitive approaches for health data collection, or digital access strategies for hard-to-reach target populations), the design of digital interventions for public health (such as applications based on artificial intelligence (AI) for research, participatory and user-centered design, frameworks for intervention design, or applying nudging methods), or the evaluation of DiPH interventions (including qualitative and quantitative methods, health technology assessments (HTA), the development of new evaluation methods suitable for the digital environment, and defining quality criteria for empirical research). While HTA was also recognized as important in the domain of health economics, this teaching content focuses on its importance for methods training. Aside from the content presented above, experts also agreed upon general teaching content, such as confidently working with statistical software (e.g., STATA, SPSS, or R), applying bibliographic and reference systems (such as EndNote, RefWorks, or ProCite), and using Boolean search methods for structured literature searches online.

Another 14 teaching contents were clustered under “Fields of application of digital interventions in healthcare,” with an average rating of 7.00–7.94. These can be grouped in innovative healthcare (i.e., using wearables and sensor data for health prediction and health promotion, applying digital tools for promoting interprofessional and interdisciplinary care concepts, or the use of AI and big data in healthcare) and digital intervention types and system change (such as patient-centered healthcare applications in simple languages, remote care through telehealth and telemedicine, digital clinical decision support tools, or the establishment of health information systems).

The third-most-named core teaching content (nine in total) was consolidated into the topic area “Health communication,” with an average vote of 7.00–8.06. These can be clustered in the design and development of health information (e.g., evidence-based, culturally appropriate and lay-friendly, as well as opportunities for automated communication between healthcare stakeholders and evaluation of chatbot output for health information communication), the use of different communication channels, including social media (for science communication and health information communication purposes), and health-related misinformation (including “fake news”).

Further, eight core teaching contents were rated as important in the topic area “Health promotion, education, and prevention,” with an average rating of 7.35–7.89. This topic area included one teaching content (“(Digital) health promotion in (digitalized) living environments”) which, depending on where to place the brackets, either describes the use of digital approaches for health promotion in living environments in general (guided by the question “How can we reach the target population digitally?”) or the application of health promotion approaches for digitalized living environments (following the question “How can we reach the target population online?”). Due to its inclusive structure, this teaching content serves as a roof, encompassing the other, more specific teaching content. Other teaching contents within this topic area targeted vulnerable populations (e.g., digital target group-specific measures and access strategies for health promotion and prevention, as well as strategies for health promotion and prevention in digital (living) environments) and digital measures (for health promotion and prevention, as well as their relevance in healthcare).

Seven items were identified as important in the topic area “Health policy and systems,” with an average vote of 7.04–7.53. Here, experts highlighted the system level of the digital transformation (e.g., changes in the healthcare system through digitization, how to strengthen resilience and responsiveness in emergency situations through digital strategies, setting up and components of a telematics infrastructure, and international comparison of digitalized healthcare systems), health data (e.g., the application of findable, accessible, interoperable, and reusable principles (FAIR) for health data (21) and data-informed policy), and the use of digitalization to ensure access to and information about healthcare services among the general public.

A sixth category, “Determinants of health, illness, and social inequalities”, included seven additional teaching contents. These received an average vote of 7.11- 8.03 on the Likert scale and targeted literacy (e.g., the definition, understanding, analysis, and promotion of digital health literacy as well as digital and information technology literacy), the digital divide (such as the definition and forms of the digital divide or the influence of digital innovations on health and social inequality), the acceptance of digital health services among the population, or the general risks and opportunities of digitalization in health and disease for the individual user and the population.

Another domain, “Ethics and law”, consisted of six core teaching contents. Here, the average consensus ranged from 7.17–7.57. This included regulation (e.g., legal requirements for human-technology innovations, security and confidentiality of health data through data protection laws, or the implication of the General Data Protection Regulation by the European Union for the German healthcare system) and ethics (i.e., unethical behavior and social responsibility when using digital health applications or health data or bioethical issues of digitalization including patient sovereignty in remote healthcare, nudging and behavioral manipulation, or the use of algorithms in health applications).

Five teaching contents were classified as “Epidemiology,” with an average rating of 7.03–7.74. These consist of content related to infodemiology (e.g., text analysis and natural language processing, search engine analysis, web scraping and data mining, or social media analytics) and digital approaches to enhance classic epidemiology (e.g., general computer-assisted methods for epidemiology, tracking and containing infectious diseases via health data, digital surveillance, or epidemiological modeling).

Five additional content items related to the topic area “IT and technology” received on average 7.16–7.47 points. Here, experts highlighted the need to educate students on interoperability (including organizational, semantic, and structural interoperability in digitalized healthcare systems) and user-friendly design (including overcoming technical access barriers for patients and universal design for digital health services that follow human-computer interaction principles).

Another five content items related to “Health economics and management,” with an average rating of 7.26–8.09. This included HTA (e.g., the application and use of ethical, social, and legal issues (ELSI) for HTA (21), and health economic evaluations as well as quality standards for health technologies) and the implementation of digital health services (focusing on their integration into existing work flows among healthcare providers and the public health workforce as well as change management).

The final set of teaching contents targeted “Public health and social medicine” from a theory-driven perspective. Here, experts ranked four teaching contents with an average of 7.44–7.97 on essential concepts for DiPH (including data science in medicine, digital health, digital public health, electronic health, and mobile health), an overview of application fields and structures for digital healthcare in health sciences, and the use of digital strategies to support clinical trials and population-level studies.

### 3.3 Most important teaching content

#### 3.3.1 Prioritization of digital public health teaching content for each topic area

**Figure 3:**
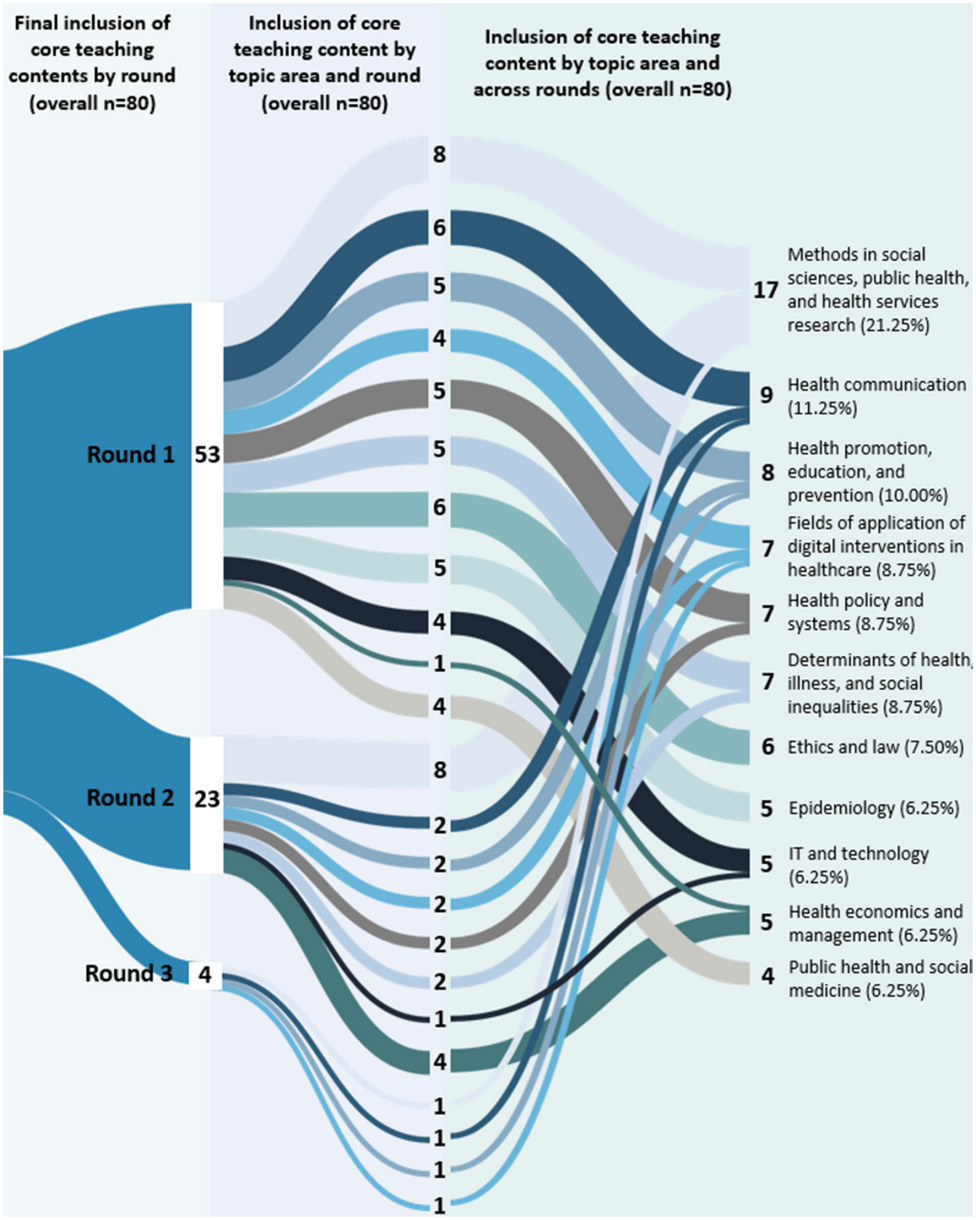
Top 3 teaching contents per public health domain.

In R3, the previously agreed teaching content (M≥7) was prioritized for each topic area based on its relevance. The teaching content that was still being voted on in R3 and ultimately not agreed upon was included in the ranking, as no decision had been made on their inclusion or exclusion at the time of the survey (*n* = 18). In total, participants ranked 103 teaching content, grouped among the 11 topic areas: Five for “Public health and social medicine”, six each for “Health economics and management” and “Epidemiology”, seven for “Determinants of health, disease, and social inequalities” as well as for “IT and technology”, eight for “Ethics and law” as well as for “Health promotion, education, and prevention”, 10 for “Health policy and healthcare systems” as well as for “Areas of application for digital interventions and tools in healthcare”, and 18 each for “Methods in social sciences” and for “Health communication”. After R3, 14 teaching contents were excluded due to missing consensus (ranging from 0 to 3 per topic area). With two exceptions (one teaching content in the topic area “Methods in the social sciences” and “Ethics and law” each), all teaching content excluded after R3 were ranked last on average. Figure 3 provides an overview of the top three teaching contents per domain, including the average rank for each content. The lower the number, the higher the teaching content was ranked by the participants. As such, a rank of 1.00 would represent the highest possible priority for each domain. The average rank for the most important teaching content per domain was “Significance and fundamentals of digital health, digital public health, electronic health, and mobile health” in “Public health and social medicine” with an average rank of 1.48, whereas the domain with the lowest average rank for the most important content was the teaching content “(Digital) health promotion in (digitalized) living environments” in the domain “Health promotion, education, and prevention” with an average rank of 3.25. This suggests that, overall, the experts were less consistent in their rankings for some domains than for others. Supplementary material 2 provides the average rank for every teaching content. All 23 experts who provided responses in R3 participated in this exercise.

#### 3.3.2 Top 10 important digital public health teaching content

In R3 of questioning, respondents selected the ten most important teaching content from all 103 teaching contents that had either already reached consensus or were still discussed as part of R3 (*n* = 18, of which 14 were excluded during R3). Unlike when prioritizing teaching content within the individual areas of public health, they were not asked to rank their top 10 teaching topics. Instead, they were asked to select the most important ones. Among the excluded 14 teaching contents, eight were selected by one participant each, and two teaching contents were selected by two experts each for the top 10. The remaining four teaching contents that were not included after R3 were not selected by any expert as among the most important overall. However, this is not significant here, as all learning contents excluded after R3 were not among the teaching contents most frequently voted into the top 10 (see Table 4, which displays the final list of the top 10 teaching content across all eleven topic areas).

**Table 4:** Teaching content most frequently voted into the top 10, *n* (%).

No.	Teaching content	Related topic area	Frequency of selection^*^
1	Participatory approaches and user orientation for digital applications (e.g., acceptance research)	Methods	9/20 (45%)
2	Development and evaluation/effectiveness measurement of digital public health interventions	Methods	9/20 (45%)
3	Significance and fundamentals of digital health, digital public health, electronic health, and mobile health	Social medicine	8/20 (40%)
4	Target group–specific health promotion and prevention in digital environments	Health promotion	6/20 (30%)
5	Artificial Intelligence and Big Data in healthcare applications	Application of digital tools	6/20 (30%)
6	Health technology assessments and health economic evaluations for digital health applications	Health economics	6/20 (30%)
7	Ethical and legal requirements for human–technology innovations	Ethics and law	6/20 (30%)
8	(Development of) evidence–based digital health information	Health communication	6/20 (30%)
9	Definition, understanding, analysis, and promotion of digital health literacy	Determinants	5/20 (25%)
10	Changes in the healthcare system through digitization strategies or the use of digital health applications	Health policy	5/20 (25%)

^*^The percentage refers to a total of 20 participants who answered the question.

Only 20 of the 23 experts who participated in R3 answered the question about the top 10 most important teaching content for a digital public health curriculum. The selected teaching content stems from nine of the eleven topic areas of digital public health, demonstrating a broad range of core teaching material. No teaching content from the fields of “Epidemiology” or “IT and technology” was among the 10 most frequently selected overall.

### 3.4 Alignment of teaching contents with established competence frameworks

In order to compare the international relevance of our findings, we conducted a content analysis of our proposed teaching contents with the ASPHER core-competence framework (10) and the Global Competence and Outcome Framework of the WHO (20). Due to the length of the ASPHER framework, we compared our findings only with the competencies outlined in the chapter *Digital Transformation in Public Health*. The complete assessment is available in Supplementary material.

In the ASPHER framework (10), all 63 proposed competences were addressed by at least one teaching content in our study. In total, we mapped 84 of our overall assessed teaching contents to the DiPH-related competencies (an average of 1.3 teaching contents per ASPHER competence), of which 54 were included in the final DiPH teaching content list after R3. For the ASPHER perspective, 23 of the 63 proposed competences were excluded from our final list of teaching contents. Four ASPHER competencies were addressed by multiple of our teaching contents, for which at least one was included, and one was excluded from the final list of competencies (see Table 5).

**Table 5:** Comparison of ASPHER core competencies in digital public health with included and excluded teaching content after the third Delphi round.

No.	ASPHER competence	Included teaching content	Excluded teaching content
1	Digital health tools (hardware and software)	Handling of appropriate hardware and software for processing health information	Hardware and software for processing information in healthcare and healthcare facilities
2	Usability across the age range	User–friendliness of digital health services across all age groups (universal design and human–computer interaction)	Design guidelines, phase, and process models of usability engineering for digital health applications
3	Use of drones for population surveillance	Digital surveillance (including health protection against infectious and non–infectious diseases)	Drones and delivery of healthcare services (medications)
4	Spread of information across digital networks	Opportunities offered by digital networking, care provision, and uniformly available data for inter–professional healthcare	(Digital) networks in healthcare: Connecting features of digitalization (freedom from space and time limits)

For the WHO framework (20), we identified 20 global competencies that were not particularly focused on DiPH or digital transformation. Nevertheless, our final list of teaching content addressed 13 of those by at least one teaching content (*n* = 31; average of 2.3 DiPH competencies per WHO competence), of which all were included in our final list. Not included were digital teaching contents surrounding the WHO global competencies of:

- Enabling people to increase control over, and to improve, their health and lives.
- Applying systems thinking to public health problem-solving.
- Adapting to unexpected or rapidly changing situations.
- Learning from, with, and about others.
- Constructively managing tensions, conflicts, resistance, and opposition.
- Working within the limits of competence and role responsibilities.
- Engaging in lifelong learning.

This might be because those competencies are generally relevant to public health education beyond the specified DiPH-related content.

## 4 Discussion

### 4.1 Discussion of results

In our Delphi study, we reached consensus on 80 teaching topics for DiPH education, spread across 11 topic areas in public health. The variety of identified teaching content displays once again the heterogeneity of public health itself. Consequently, the included content covers all essential public health functions (22). This reinforces the initial assumption that it is simply impossible to work in public health today without encountering digital tools or information (6). Hence, a well-prepared and educated future workforce will be essential to ensuring a sustainable healthcare system. As Alotaibi et al. highlighted in their review, a lack of workforce training can create a barrier to the use and acceptance of digital tools and interventions for healthcare and health promotion (23). Our participants emphasized the need for a multidisciplinary list of teaching content to be implemented across every public health teaching program, consistent with the findings of Ramachandran et al. (24). While our study is situated in the public health education context in Germany, many of the identified teaching contents reflect globally relevant challenges related to digital transformation. However, the applicability of specific content areas may vary depending on local infrastructure, workforce needs, and educational systems, particularly in low- and middle-income countries or highly digitalized health systems. In support of a global review of DiPH training programs (25), our experts reached consensus on the importance of data-centric teaching content and ranked it as a top priority across multiple public health topic areas. Contrary to this study, our experts focused particularly on teaching content on digital health promotion and the determinants of health and inequality, which Iyamu et al. (25) identified as current teaching gaps.

A closer look at the top 10 teaching contents highlights a clear emphasis on applied, practice-oriented competencies. The two highest-ranked topics (participatory approaches and user orientation, as well as the development and evaluation of digital public health interventions) reflect the importance of actively designing and assessing interventions in real-world settings. This supports existing evidence that successful digital public health implementation relies heavily on user-centered design and robust evaluation strategies (26–28). Interestingly, although AI and Big Data are widely expected to shape the future of public health, they were named by fewer participants as crucial, and therefore ranked fifth among the top 10 teaching contents. This may reflect that experts prioritized more immediately applicable, practice-oriented skills (such as participatory approaches and intervention evaluation) while recognizing that AI and Big Data competencies, though critical, build on foundational knowledge and experience. Also, no teaching content from epidemiology or IT and technology was among the top-ranked items, suggesting that these areas are perceived as foundational and already embedded in existing curricula, while the focus is shifting toward their application in digital contexts. However, this observation may also be influenced by the small proportion of participants with a main field of work in epidemiology (R1: *n* = 3; R2: *n* = 2).

Similar to the publications by Yao et al., Alotaibi et al., and Hrzic et al. (23, 29, 30), our experts advocated for in-depth knowledge and understanding of digital health literacy, as well as for developing, identifying, and using evidence-based digital health information. This also includes the emerging areas of AI literacy and the ethical use of AI tools, as noted by Hrzic et al. and Acosta (29, 31). In line with their claims, our participants highlighted the need for sufficient AI literacy and knowledge of AI and Big Data use in healthcare applications. With an increasing number of tools using large language model-components being developed and pushed onto the market [Open AI has just announced its new ChatGPT Health model in January 2026 (32)], it becomes growingly important for the public health workforce to confidently navigate across these tools and being able to critically assess their outputs for best use within public health interventions (33).

Contrary to previous studies on DiPH education, our experts highlighted the importance of participatory approaches in developing and implementing digital tools and interventions for public health purposes. This often-overlooked skill becomes increasingly important, especially in the digital environment, where adherence rates quickly drop due to tools that have not been designed to prioritize user needs (26–28). As such, our most often-referred-to teaching content among those discussed, “Participatory approaches and user orientation for digital applications (e.g., acceptance research)”, will serve as a guiding principle for the importance of co-creative skills for the future public health workforce (34).

However, it must also be noted that not all areas within public health have been highlighted in this study based on their relevance to digitalization. For instance, the development of public health policies was not included in this study, as it was neither proposed by participants nor explicitly mentioned in the DiPH-related section of the ASPHER core-competency framework (10). However, as highlighted by ASPHER (10) and the WHO Global Competency and Outcomes Framework (20), translating evidence into policy and action, advocacy, and the critical appraisal of research in public health are important topics for general public health education. Therefore, when designing or revising a public health curriculum, it is essential to critically examine each course component: Which teaching content is of such general importance to the future professional practice of graduates that it should be integrated into general public health education, and which content raises additional questions due to the digital transformation and is, as such, becoming more relevant for a designated curriculum in DiPH?

### 4.2 Strengths and limitations

Our study comes with several strengths and limitations. A key strength is the use of the Delphi method, which has proven effective in reaching consensus on complex, multidisciplinary topics, such as those encountered in DiPH training. Delphi studies ensure participant anonymity, thereby reducing the risk of bias due to social desirability. Likewise, the multi-stage survey design reduces the risk of dominant participants compared to focus group studies (11). Furthermore, we strictly adhered to best practice and reporting guidelines for Delphi studies in the social and health sciences (35), and we have provided the completed checklist for our manuscript in Supplementary material. In accordance with the quality standards for Delphi studies, both the author group and the study cohort comprise experts with extensive experience in public health and DiPH across academia, business, healthcare, and government. Participation rates were high: 50% of pre-survey registrants and 66% of R1 participants contributed to all three Delphi rounds. This underscores the perceived importance of this topic among our experts.

Nevertheless, several limitations should be considered when interpreting the results. We asked experts about their academic backgrounds, not their areas of focus within public health. This could have led to an overrepresentation of experts in specific areas of public health (e.g., health economics or health promotion), thereby potentially influencing the selection and prioritization of teaching content (e.g., epidemiology or IT). However, the final list of teaching content comprises 11 topic areas, each with between 4 and 17 teaching contents. We are therefore confident that our study has produced a balanced list of teaching contents that should be considered for general public health education. Because the study is conducted exclusively with German experts on the German public health education system, there is a risk that some teaching content may not be applicable to other settings outside Germany. However, as our findings are consistent with those of other international studies, we are convinced that our study nevertheless has international relevance. Additionally, our study reflects only the perspectives of public health experts across various settings and does not capture those of other key stakeholders, such as students, employers, or policymakers. Including them could lead to different priorities, thereby influencing the selection and ranking of competencies.

Another limitation is that experts in the pre-survey were not asked to rank the teaching content, resulting in a loss of votes from six experts in R1. This decision was made to avoid overburdening the participants due to the length of the first two surveys. While methodologically justified, this could have influenced which items reached consensus and their eventual ranking, adding some uncertainty to the robustness of the final top 10. Further, it must be noted that the inclusion criterion of at least 7 out of 9 points on average on the Likert scale for teaching contents was chosen arbitrarily, even though best-practice guidelines were considered and other Delphi approaches were examined for guidance (35, 36). While a stricter threshold criterion might have led to more teaching content being excluded (all excluded content were no longer considered due to them being presented twice without finding consensus on their importance or lack of importance for a DiPH curriculum), the final list of teaching contents displays the heterogeneity of DiPH and public health well, while allowing for a broad and a specialized range of knowledge (T-shaped), making it perfectly applicable to the ideal public health professional that Iyamu et al. pointed out as essential for future workforce profiles (25). Finally, it must be noted that the combination of R3 (consensus) and the ranking of teaching content that has already been agreed upon and presented (again) in R3 may have introduced distortions. The decision was made due to declining participant numbers from R1 to R2 (23% drop-out), as it was considered too risky to lose more participants in R4, which would have negatively impacted the ranking exercises. Further, it should be noted that, with the exception of two teaching topics, all 14 excluded topics ranked at the bottom of each topic area, and none made it into the overall top 10 of teaching content.

## 5 Conclusion

Our current study has well described how DiPH should be taught as part of the general public health education and which topics are essential to consider. Together with our underlying analysis of DiPH-specific modules taught in public health education in Germany (9), we have demonstrated what DiPH education could look like: it requires multidisciplinary perspectives and input across various domains, shaped by the digital transformation in public health. Most importantly on this behalf are the education of students in co-creative approaches, the evaluation of digital interventions in health and public health together with HTAs, terminological clarity between the most dominantly used terms, the application of AI and big data for healthcare, digitalized target-group specific health promotion strategies, an understanding of ethical and legal principles for health data, digital health literacy, and changes in the healthcare system due to the digital transformation.

Although based on expert consensus rather than empirical evaluation, the findings may inform curriculum development and policy discussions and provide a basis for future competency-based frameworks in DiPH. While not the aim of this study, the identified and prioritized teaching contents can support the systematic, theory-informed definition of competencies and learning objectives. As such, educational programs might consider integrating experiential and practice-oriented modules, and accreditation bodies could reflect on whether current learning objectives adequately address emerging digital competencies.

Future research is needed to assess the current status of DiPH education from the perspectives of public health program managers regarding implementation barriers to digital skills in curricula, as well as from students regarding their sense of being sufficiently prepared for the digital transformation of health and public health. Further research is needed to bring academia together with potential future employers, such as industry, ministries, public health agencies, and health insurance, to assess whether current public health education meets the job-market requirements for graduates in terms of digital skills. Only by placing greater focus on actual groundwork can we identify gaps and overcome implementation barriers to truly prepare our future workforce for a setting where digital skills are an essential part of every task related to promoting a healthy population.

## Data Availability

The original contributions presented in the study are included in the article/Supplementary material, further inquiries can be directed to the corresponding author.
