# Supplementary material for: Building consensus on core teaching content of a digital public health curriculum: a Delphi study with public health experts in Germany
Source: Front Public Health. 2026 Jun 19;14:1799393. doi: 10.3389/fpubh.2026.1799393 (PMC13328418; doi:10.3389/fpubh.2026.1799393)
Supplement: Supplementary file 1 [file Table_1.docx]

# Supplementary Material 1: Core teaching content for digital public health

## Table A1: Overview of teaching content agreed upon as important (included)

| **No.** | **Topic area** | **Final teaching content that was agreed upon as important** | **Mean R1** | **Mean R2** | **Mean R3** | **Direct**  **Inclusion**  **(after 1st presentation)** | **Indirect**  **Inclusion**  **(after 2nd presentation)** |
| --- | --- | --- | --- | --- | --- | --- | --- |
| 1 | Fields of application of digital interventions in healthcare | Digital applications for promoting interprofessional healthcare as part of innovative, interdisciplinary care concepts | - | - | 7.05 | x |  |
| 2 |  | Artificial Intelligence and Big Data in healthcare applications | 7.94 | - | - | x |  |
| 3 |  | Remote care, telehealth and telemedicine | 7.38 | - | - | x |  |
| 4 |  | Health information systems | 7.33 | - | - | x |  |
| 5 |  | Wearables and sensors for health prediction and health promotion | 7.12 | - | - | x |  |
| 6 |  | Patient-centered healthcare applications and educational offerings in simple language | - | 7.15 | - | x |  |
| 7 |  | Digital clinical decision support tools | - | 7.00 | - | x |  |
| 8 | Health communication | (Development of) evidence-based digital health information | 8.06 | - | - | x |  |
| 9 |  | Health communication via digital and social media | 7.60 | - | - | x |  |
| 10 |  | Design of culturally appropriate and layperson-friendly digital health information and services | 7.59 | - | - | x |  |
| 11 |  | Use of digital communication channels (computer-based, web-based, multimedia processes) | 7.40 | - | - | x |  |
| 12 |  | Science communication via digital and social media | 7.09 | - | - | x |  |
| 13 |  | Health-related misinformation (misinformation, “fake news”) | - | 7.48 | - | x |  |
| 14 |  | Science communication on research with health data and the transfer of scientific findings into practice | - | 7.27 | - | x |  |
| 15 |  | Evaluation of the communication quality of digital applications (e.g., chatbots) between healthcare providers (e.g., physicians) and patients | - | 6.89 | 7.00 |  | x |
| 16 |  | Automation of communication processes and collaboration between healthcare stakeholders | 7.29 | - | - | x |  |
| 17 | Health economics and management | Health technology assessments and health economic evaluations for digital health applications | 8.09 | - | - | x |  |
| 18 |  | Quality criteria and standards for health technologies | - | 7.81 | - | x |  |
| 19 |  | Implementation of health management processes using digital services and applications: promoting acceptance, user-friendliness, and change management | - | 7.54 | - | x |  |
| 20 |  | Application and use of ELSI issues (ethical, social, and legal issues in health technology assessments) | - | 7.48 | - | x |  |
| 21 |  | Integration of digital services and applications into existing work processes | - | 7.26 | - | x |  |

| **No.** | **Topic area** | **Final teaching content that was agreed upon as important** | **Mean R1** | **Mean R2** | **Mean R3** | **Direct**  **Inclusion**  **(after 1st presentation)** | **Indirect**  **Inclusion**  **(after 2nd presentation)** |
| --- | --- | --- | --- | --- | --- | --- | --- |
| 22 | Epidemiology | Strategies for tracking and containing pathogens and infectious diseases using health data | 7.74 | - | - | x |  |
| 23 |  | Use of digital or computer-assisted methods in epidemiology | 7.67 | - | - | x |  |
| 24 |  | Digital surveillance (including health protection against infectious and non-infectious diseases) | 7.42 | - | - | x |  |
| 25 |  | Modeling methods for epidemics of infectious and non-infectious diseases and environmental changes | 7.24 | - | - | x |  |
| 26 |  | Infodemiology (e.g., text analysis and natural language processing, search engine analysis, web scraping and data mining, or social media analytics) | 7.03 | - | - | x |  |
| 27 | Ethics and law | Ethical and legal requirements for human-technology innovations | 7.57 | - | - | x |  |
| 28 |  | Unethical behavior and social responsibility in the use of digital applications (in relation to vulnerable groups) | 7.54 | - | - | x |  |
| 29 |  | General guarantee of security and confidentiality of health data | 7.51 | - | - | x |  |
| 30 |  | Ethical use and implications of health data and management (e.g., consequences of data analysis and interpretation) | 7.47 | - | - | x |  |
| 31 |  | Data protection laws and national laws on health data (e.g., the significance of the GDPR for Germany) | 7.46 | - | - | x |  |
| 32 |  | Bioethical issues of digitalization (e.g., the use of Artificial Intelligence, Big Data, and algorithms in healthcare, patient sovereignty in telemedicine, nudging and behavioral manipulation, etc.) | 7.17 | - | - | x |  |
| 33 | Determinants of health, illness, and social inequalities | Definition, understanding, analysis, and promotion of digital health literacy | 8.03 | - | - | x |  |
| 34 |  | The relationship between the use of digital innovations and (the reduction or increase in) health and social inequality | 7.91 | - | - | x |  |
| 35 |  | Definition, understanding, analysis, and promotion of digital and information technology literacy | 7.72 | - | - | x |  |
| 36 |  | Analysis of the acceptance of digital health services among the population | 7.71 | - | - | x |  |
| 37 |  | Definition and introduction to the digital divide: forms and models of the digital divide | - | 7.17 | - | x |  |
| 38 |  | Acceptance and willingness to use digital health services among the population (distribution by population groups and influencing factors) | - | 7.11 | - | x |  |
| 39 |  | Information about the risks and opportunities of digitization in health and disease | 7.71 | - | - | x |  |
| 40 | IT and technology | Organizational interoperability (effective collaboration and communication between different organizations within the system despite differing structures and processes) | 7.31 | - | - | x |  |
| 41 |  | Semantic interoperability (application of standardized terminologies, classification systems, and data formats to ensure that exchanged data is understood and interpreted in the same way by the systems and organizations involved) | 7.25 | - | - | x |  |
| 42 |  | Structural interoperability (exchange of data in the correct format and structure between systems and organizations via standardized data formats and communication protocols) | 7.16 | - | - | x |  |

| **No.** | **Topic area** | **Final teaching content that was agreed upon as important** | **Mean R1** | **Mean R2** | **Mean R3** | **Direct**  **Inclusion**  **(after 1st presentation)** | **Indirect**  **Inclusion**  **(after 2nd presentation)** |
| --- | --- | --- | --- | --- | --- | --- | --- |
| 43 | IT and technology | User-friendliness of digital health services across all age groups (universal design and human-computer interaction) | 7.47 | - | - | x |  |
| 44 |  | Technical barriers to access for patients (e.g., people with limited physical/mental abilities) | - | 7.22 | - | x |  |
| 45 | Methods in the social sciences | Opportunities offered by digital networking, care provision, and uniformly available data for interprofessional healthcare | - | - | 7.04 | x |  |
| 46 |  | Methods for collecting and evaluating health data | 8.14 | - | - | x |  |
| 47 |  | Quantitative and qualitative methods for digital health topics | 8.09 | - | - | x |  |
| 48 |  | Participatory approaches and user orientation for digital applications (e.g., acceptance research) | 8.00 | - | - | x |  |
| 49 |  | Assessment of health technologies (assessment of technological consequences) | 7.66 | - | - | x |  |
| 50 |  | Proficiency in statistical software (e.g., STATA, SPSS, SAS, R) | 7.56 | - | - | x |  |
| 51 |  | Understanding and executing appropriate Boolean search methods | 7.44 | - | - | x |  |
| 52 |  | Methods of diversity-sensitive data collection | 7.38 | - | - | x |  |
| 53 |  | Handling of appropriate hardware and software for processing health information | 7.29 | - | - | x |  |
| 54 |  | Access strategies for hard-to-reach target groups in digital health research | - | 8.08 | - | x |  |
| 55 |  | Dealing with AI-based applications in public health research | - | 7.78 | - | x |  |
| 56 |  | Quality criteria for digital empirical health research | - | 7.77 | - | x |  |
| 57 |  | Procedural models for developing frameworks for (complex) digital interventions | - | 7.70 | - | x |  |
| 58 |  | Working with bibliographic and referencing systems (e.g., EndNote, Reference Manager, Refwork, and Procite) | - | 7.50 | - | x |  |
| 59 |  | Use of new sources for health data (e.g., social media or wearables) | - | 7.35 | - | x |  |
| 60 |  | Application of nudging methods in the intervention design of digital health applications | - | 7.22 | - | x |  |
| 61 |  | Development and evaluation/effectiveness measurement of digital public health interventions | - | 7.09 | - | x |  |
| 62 | Health policy and systems | Changes in the healthcare system through digitization strategies or the use of digital health applications | 7.53 | - | - | x |  |
| 63 |  | Use of health data and information technologies for policy makers (data-informed policy) | 7.46 | - | - | x |  |
| 64 |  | Creating access to healthcare services and information through digitalization | 7.37 | - | - | x |  |
| 65 |  | Strengthening the resilience and responsiveness of the healthcare system in emergency situations through digital strategies and applications (e.g., early warning systems, AI-supported triage tools, training, etc.) | 7.29 | - | - | x |  |
| 66 |  | Telematics infrastructure in healthcare | 7.29 | - | - | x |  |
| 67 |  | International comparison of healthcare systems with regard to the digitization of healthcare systems | - | 7.22 | - | x |  |
| 68 |  | Organization, management, and control of health data (FAIR principles) | - | 7.04 | - | x |  |

| **No.** | **Topic area** | **Final teaching content that was agreed upon as important** | **Mean R1** | **Mean R2** | **Mean R3** | **Direct**  **Inclusion**  **(after 1st presentation)** | **Indirect**  **Inclusion**  **(after 2nd presentation)** |
| --- | --- | --- | --- | --- | --- | --- | --- |
| 69 | Health promotion, education, and prevention | Social space-oriented digital care and prevention concepts | - | - | 7.35 | x |  |
| 70 |  | Digital measures for health promotion | 7.89 | - | - | x |  |
| 71 |  | Digital prevention measures | 7.76 | - | - | x |  |
| 72 |  | (Digital) health promotion in (digitalized) living environments | 7.56 | - | - | x |  |
| 73 |  | Relevance of digital prevention approaches in healthcare | 7.56 | - | - | x |  |
| 74 |  | Media strategies for health promotion (e.g., social media) | 7.43 | - | - | x |  |
| 75 |  | Digital target group-specific measures and access strategies for health promotion and prevention (with a particular focus on vulnerable groups) | - | 7.63 | - | x |  |
| 76 |  | Target group-specific health promotion and prevention in digital environments (with a particular focus on vulnerable groups) | - | 7.54 | - | x |  |
| 77 | Public health and social medicine | Digital application fields and care structures in health sciences | 7.97 | - | - | x |  |
| 78 |  | Significance and fundamentals of digital health, digital public health, electronic health, and mobile health | 7.91 | - | - | x |  |
| 79 |  | Digital strategies and applications to support clinical trials and population-level studies | 7.56 | - | - | x |  |
| 80 |  | Data science in medicine | 7.44 | - | - | x |  |
| **Total Number** | | | | | | **79** | **1** |

## Table A2: Overview of teaching content without agreement on importance (excluded)

| **No.** | **Topic area** | **Final teaching content that was agreed upon as important** | **Mean R1** | **Mean R2** | **Mean R3** | **Direct**  **Exclusion**  **(after 1st presentation)** | **Indirect**  **Exclusion**  **(after 2nd presentation)** |
| --- | --- | --- | --- | --- | --- | --- | --- |
| 1 | Fields of application of digital interventions in healthcare | (Digital) networks in healthcare: Connecting features of digitalization (freedom from space and time limits) | - | 6.87 | 6.71 |  | x |
| 2 |  | Blockchain and distributed ledger technologies in electronic patient records | 6.26 | 6.19 | - |  | x |
| 3 |  | Digital management of vaccination appointments | 6.25 | 6.92 | - |  | x |
| 4 |  | Digital management of medications | 6.29 | 5.91 | - |  | x |
| 5 |  | Drones and delivery of healthcare services (medications) | 5.40 | 6.23 | - |  | x |
| 6 |  | Genomics (applications and self-testing mechanisms) | 6.41 | 5.42 | - |  | x |
| 7 |  | Health counseling in peer groups with AI support | - | 6.27 | 5.68 |  | x |
| 8 |  | Hardware and software for processing information in healthcare and healthcare facilities | 6.78 | 5.15 | - |  | x |
| 9 |  | Medication calculators | 6.13 | 6.23 | - |  | x |
| 10 |  | Pathology and X-ray software | 5.69 | 5.84 | - |  | x |
| 11 |  | Robotics (medicine, surgery and pharmacy) | 6.23 | 5.85 | - |  | x |
| 12 |  | Software-supported care documentation (e.g., via telecare, DiGA, DiPA, care robotics, chatbots, or computerized care plans) | 6.52 | 6.58 | - |  | x |
| 13 |  | Virtual and augmented reality in medical and public health education, training, and continuing education | - | 6.65 | 6.14 |  | x |
| 14 |  | Virtual and augmented reality in healthcare applications | 6.88 | 6.81 | - |  | x |
| 15 | Health communication | Evaluation of health communication strategies in digital media using communication science methods | 6.85 | 5.08 | - |  | x |
| 16 |  | Digital risk communication on food safety | 5.85 | 6.44 | - |  | x |
| 17 | Health economics and management | Sustainable financing models for digitally supported services and applications (e.g., financing health tech start-ups) | - | 6.63 | 6.09 |  | x |
| 18 |  | Personalization and precision of digital public health services (e.g., digital twins) | 6.65 | 6.88 | - |  | x |
| 19 |  | Restructuring of work processes to take online downtime into account | 5.76 | 5.04 | - |  | x |
| 20 | Epidemiology | Identification, evaluation, and use of secondary data (e.g., Google searches, biostatistics, etc.) | - | 6.85 | 6.86 |  | x |
| 21 |  | Modeling of population dynamics | 6.88 | 6.41 | - |  | x |
| 22 |  | Use of software for calculating epidemiological study cohorts (e.g., Plug & Chug Epi Calculator, EpiTab, OpenEpi, WinPEPI, PS, etc.) | 6.77 | 6.64 | - |  | x |
| 23 |  | Use of GPS and proximity data (e.g., in pandemics) | 6.76 | 6.77 | - |  | x |

| **No.** | **Topic area** | **Final teaching content that was agreed upon as important** | **Mean R1** | **Mean R2** | **Mean R3** | **Direct**  **Exclusion**  **(after 1st presentation)** | **Indirect**  **Exclusion**  **(after 2nd presentation)** |
| --- | --- | --- | --- | --- | --- | --- | --- |
| 24 | Ethics and law | Application of the MEESTAR model for the ethical evaluation of socio-technical arrangements (instrument of applied ethics for concrete technical applications in practice) | - | 6.44 | 6.24 |  | x |
| 25 |  | Moral issues in technology development and use (technical ethics) | 6.77 | 6.74 | - |  | x |
| 26 |  | Regulations according to the Medical Device Regulation (MDR) for the classification and certification of digital medical devices | - | 6.30 | 6.32 |  | x |
| 27 | IT and technology | Design guidelines, phase, and process models of usability engineering for digital health applications (a process that runs parallel to traditional planning and development work to ensure the subsequent usability of a system) | - | 6.09 | 6.29 |  | x |
| 28 |  | Information security in the healthcare industry | 6.88 | 6.52 | - |  | x |
| 29 |  | Possibilities and limitations of IT applications in healthcare facilities | 6.73 | 6.33 | - |  | x |
| 30 |  | Risk management and risk communication in healthcare (e.g., cybersecurity, backup solutions from healthcare providers, and data leaks) | 6.82 | 6.09 | - |  | x |
| 31 |  | Syntactic interoperability (formally used data formats, transmission techniques, and information models between systems) | - | 6.92 | 6.42 |  | x |
| 32 |  | Telecommunications, fundamentals of network technologies (e.g., the Internet), and principles of data transmission/security | 6.47 | 6.00 | - |  | x |
| 33 |  | Handling IT systems, programs, and databases in healthcare | 6.79 | 6.41 | - |  | x |
| 34 |  | Reuse of digital technologies (sustainability, avoidance, and reduction of duplicate structures, adaptation of existing digital services) | 6.74 | 6.85 | - |  | x |
| 36 | Methods in the social sciences | Use of data entry programs (e.g., Microsoft ACCESS, EpiData) | 6.65 | 6.48 | - |  | x |
| 36 |  | Use of data transfer software (e.g., StatTransfer) | 6.59 | 5.88 | - |  | x |
| 37 |  | Use of multi-method systems (referencing, plagiarism, and grammar systems) | 6.00 | 5.84 | - |  | x |
| 38 |  | Use of software for computer-assisted evaluation of qualitative data and text analysis (e.g., MAXQDA and F4) | - | 6.78 | 6.91 |  | x |
| 39 | Health policy and systems | The role of big tech companies in public health | - | 6.52 | 6.00 |  | x |
| 40 |  | Futures 2030 Commission (purpose, goals, milestones, etc.) | - | 5.94 | 5.50 |  | x |
| 41 |  | Fundamentals of information technologies in the healthcare system (e.g., significance, areas of application, history) | 6.29 | 6.04 | - |  | x |
| 42 |  | Overview of investments in the technical infrastructure of the healthcare system | 6.77 | 6.07 | - |  | x |
| 43 |  | Overview of relevant interest groups in the digital healthcare system | 5.97 | 5.85 | - |  | x |
| 44 |  | WHO sources and resources for the digital transformation of healthcare systems | - | 6.67 | 6.62 |  | x |
| 45 |  | Certification of digital public health services | 6.49 | 6.54 | - |  | x |
| 46 | Public health and social medicine | Definitions, history, and introduction to health informatics | - | 5.77 | 5.32 |  | x |
| 47 |  | Definitions, history, and introduction to medical informatics | 6.36 | 4.69 | - |  | x |
| 48 |  | Definitions, history, and introduction to medical technology | 4.66 | 5.15 | - |  | x |
| **Total Number** | | | | | | **0** | **48** |
