# Supplementary material for: Building consensus on core teaching content of a digital public health curriculum: a Delphi study with public health experts in Germany
Source: Front Public Health. 2026 Jun 19;14:1799393. doi: 10.3389/fpubh.2026.1799393 (PMC13328418; doi:10.3389/fpubh.2026.1799393)
Supplement: Supplementary file 2 [file Table_2.docx]

# Supplementary Material 2: Prioritization of digital public health teaching content for each topic area

## Table A3: Average ranking of teaching content in “Health economics and health management”

| **Rank** | **Teaching content** | **Mean rank** |
| --- | --- | --- |
| 1 | Quality criteria and standards for health technologies | 2.57 |
| 2 | Health technology assessments and health economic evaluations for digital health applications | 3.05 |
| 3 | Integration of digital services and applications into existing work processes | 3.26 |
| 4 | Application and use of ELSI issues (ethical, social, and legal issues in health technology assessments) | 3.63 |
| 5 | Implementation of health management processes using digital services and applications: promoting acceptance, user-friendliness, and change management | 3.94 |
| 6 | Sustainable financing models for digitally supported services and applications (e.g., financing of health tech start-ups)* | 5.71 |

*This teaching content was voted on in the third round and was ultimately not approved.

## Table A4: Average ranking of teaching content in “Methods in the social sciences”

| **Rank** | **Teaching content** | **Mean rank** |
| --- | --- | --- |
| 1 | Development and evaluation/effectiveness measurement of digital public health interventions | 2.85 |
| 2 | Assessment of health technologies (assessment of technological consequences) | 5.06 |
| 3 | Methods for collecting and evaluating health data | 5.15 |
| 4 | Participatory approaches and user orientation for digital applications (e.g., acceptance research) | 6.22 |
| 5 | Quantitative and qualitative methods for digital health topics | 6.78 |
| 6 | Quality criteria for digital empirical health research | 7.00 |
| 7 | Access strategies for hard-to-reach target groups in digital health research | 7.83 |
| 8 | Procedural models for developing frameworks for (complex) digital interventions | 9.29 |
| 9 | Methods of diversity-sensitive data collection | 9.31 |
| 10 | Dealing with AI-based applications in public health research | 9.55 |
| 11 | Opportunities offered by digital networking, care provision, and uniformly available data for interprofessional healthcare | 9.71 |
| 12 | Use of new sources for health data (e.g., social media or wearables) | 9.89 |
| 13 | Handling of appropriate hardware and software for processing health information | 10.81 |
| 14 | Proficiency in statistical software (e.g., STATA, SPSS, SAS, R) | 10.94 |
| 15 | Proficiency in using software for computer-assisted evaluation of qualitative data and text analysis (e.g., MAXQDA and F4)* | 11.06 |
| 16 | Understanding and executing appropriate Boolean search methods | 11.83 |
| 17 | Application of nudging methods in the intervention design of digital health applications | 11.93 |
| 18 | Working with bibliographic and referencing systems (e.g., EndNote, Reference Manager, Refwork, and Procite) | 13.80 |

*This teaching content was voted on in the third round and was ultimately not approved.

## Table A5: Average ranking of teaching content in “Public health and social medicine”

| **Rank** | **Teaching content** | **Mean rank** |
| --- | --- | --- |
| 1 | Significance and fundamentals of digital health, digital public health, electronic health, and mobile health | 1.48 |
| 2 | Digital application fields and care structures in health sciences | 2.10 |
| 3 | Data science in medicine | 3.32 |
| 4 | Digital strategies and applications to support clinical trials and population-level studies | 3.80 |
| 5 | Definitions, history, and introduction to health informatics* | 4.06 |

*This teaching content was voted on in the third round and was ultimately not approved.

## Table A6: Average ranking of teaching content in “Ethics and law”

| **Rank** | **Teaching content** | **Mean rank** |
| --- | --- | --- |
| 1 | General guarantee of security and confidentiality of health data | 2.53 |
| 2 | Data protection laws and national laws on health data (e.g., the significance of the GDPR for Germany) | 3.00 |
| 3 | Ethical use and implications of health data and management (e.g., consequences of data analysis and interpretation) | 3.38 |
| 4 | Bioethical issues of digitalization (e.g., the use of Artificial Intelligence, Big Data, and algorithms in healthcare, patient sovereignty in telemedicine, nudging and behavioral manipulation, etc.) | 3.85 |
| 5 | Ethical and legal requirements for human-technology innovations | 3.89 |
| 6 | Regulations according to the Medical Device Regulation for the classification and certification of digital medical devices* | 5.25 |
| 7 | Unethical behavior and social responsibility in the use of digital applications (in relation to vulnerable groups) | 5.27 |
| 8 | Application of the MEESTAR model for the ethical evaluation of socio-technical arrangements (instrument of applied ethics for concrete technical applications in practice)* | 6.50 |

*This teaching content was voted on in the third round and was ultimately not approved.

## Table A7: Average ranking of teaching content in “Areas of application for digital interventions and tools in healthcare”

| **Rank** | **Teaching content** | **Mean rank** |
| --- | --- | --- |
| 1 | Remote care, telehealth and telemedicine | 3.11 |
| 2 | Digital clinical decision support tools | 3.26 |
| 3 | Health information systems | 3.31 |
| 4 | Artificial Intelligence and Big Data in healthcare applications | 3.63 |
| 5 | Patient-centered healthcare applications and educational offerings in simple language | 5.29 |
| 6 | Wearables and sensors for health prediction and health promotion | 5.35 |
| 7 | Digital applications for promoting interprofessional healthcare as part of innovative, interdisciplinary care concepts | 5.47 |
| 8 | (Digital) networks in healthcare: Connecting features of digitalization (freedom from space and time limits)* | 5.86 |
| 9 | Virtual and augmented reality in medical and public health education, training, and continuing education* | 8.13 |
| 10 | Health counseling in peer groups with AI support* | 8.14 |

*This teaching content was voted on in the third round and was ultimately not approved.

## Table A8: Average ranking of teaching content in “Health policy and healthcare system”

| **Rank** | **Teaching content** | **Mean rank** |
| --- | --- | --- |
| 1 | International comparison of healthcare systems with regard to the digitization of healthcare systems | 3.11 |
| 2 | Use of health data and information technologies for policy makers (data-informed policy) | 3.56 |
| 3 | Changes in the healthcare system through digitization strategies or the use of digital health applications | 4.06 |
| 4 | Telematics infrastructure in healthcare | 4.16 |
| 5 | Creating access to healthcare services and information through digitalization | 4.25 |
| 6 | Organization, management, and control of health data (FAIR principles) | 4.39 |
| 7 | Strengthening the resilience and responsiveness of the healthcare system in emergency situations through digital strategies and applications (e.g., early warning systems, AI-supported triage tools, training, etc.) | 5.50 |
| 8 | WHO resources and support for the digital transformation of health systems* | 6.88 |
| 9 | The role of big tech companies in public health* | 8.08 |
| 10 | Futures 2030 Commission (purpose, goals, milestones, etc.)* | 8.29 |

*This teaching content was voted on in the third round and was ultimately not approved.

## Table A9: Average ranking of teaching content in “Health promotion, education, and prevention”

| **Rank** | **Teaching content** | **Mean rank** |
| --- | --- | --- |
| 1 | (Digital) health promotion in (digitalized) living environments | 3.25 |
| 2 | Target group-specific health promotion and prevention in digital environments (with a particular focus on vulnerable groups) | 3.68 |
| 3 | Digital target group-specific measures and access strategies for health promotion and prevention (with a particular focus on vulnerable groups) | 3.75 |
| 4 | Digital measures for health promotion | 3.79 |
| 5 | Relevance of digital prevention approaches in healthcare | 3.89 |
| 6 | Digital prevention measures | 4.32 |
| 7 | Social space-oriented digital care and prevention concepts | 5.48 |
| 8 | Media strategies for health promotion (e.g., social media) | 6.22 |

## Table A10: Average ranking of teaching content in “Determinants of health, disease, and social inequalities”

| **Rank** | **Teaching content** | **Mean rank** |
| --- | --- | --- |
| 1 | Definition, understanding, analysis, and promotion of digital health literacy | 2.76 |
| 2 | Definition and introduction to the digital divide: forms and models of the digital divide | 3.24 |
| 3 | Information about the risks and opportunities of digitization in health and disease | 3.58 |
| 4 | Definition, understanding, analysis, and promotion of digital and information technology literacy | 3.59 |
| 5 | The relationship between the use of digital innovations and (the reduction or increase in) health and social inequality | 3.67 |
| 6 | Acceptance and willingness to use digital health services among the population (distribution by population groups and influencing factors) | 3.83 |
| 7 | Analysis of the acceptance of digital health services among the population | 4.63 |

## Table A11: Average ranking of teaching content in 10 “IT and technology”

| **Rank** | **Teaching content** | **Mean rank** |
| --- | --- | --- |
| 1 | User-friendliness of digital health services across all age groups (universal design and human-computer interaction) | 2.58 |
| 2 | Semantic interoperability | 3.35 |
| 3 | Organizational interoperability | 3.44 |
| 4 | Technical barriers to access for patients (e.g., people with limited physical/mental abilities) | 3.82 |
| 5 | Structural interoperability | 4.00 |
| 6 | Design guidelines, phase and process models of usability engineering for digital health applications* | 4.06 |
| 7 | Syntactic interoperability* | 4.71 |

*This teaching content was voted on in the third round and was ultimately not approved.

## Table A12: Average ranking of teaching content in “Epidemiology”

| **Rank** | **Teaching content** | **Mean rank** |
| --- | --- | --- |
| 1 | Digital surveillance (including health protection against infectious and non-infectious diseases) | 2.20 |
| 2 | Use of digital or computer-assisted methods in epidemiology | 2.26 |
| 3 | Strategies for tracking and containing pathogens and infectious diseases using health data | 3.15 |
| 4 | Modeling methods for epidemics of infectious and non-infectious diseases and environmental changes | 3.72 |
| 5 | Infodemiology (e.g., text analysis and natural language processing, search engine analysis, web scraping and data mining, or social media analytics) | 4.29 |
| 6 | Identification, evaluation, and use of secondary data (e.g., Google searches, biostatistics, etc.)* | 4.29 |

*This teaching content was voted on in the third round and was ultimately not approved.

## Table A13: Average ranking of teaching content in “Health communication”

| **Rank** | **Teaching content** | **Mean rank** |
| --- | --- | --- |
| 1 | (Development of) evidence-based digital health information | 2.50 |
| 2 | Health communication via digital and social media | 3.05 |
| 3 | Use of digital communication channels (computer-based, web-based, multimedia processes) | 4.44 |
| 4 | Health-related misinformation (misinformation, “fake news”) | 4.63 |
| 5 | Design of culturally appropriate and layperson-friendly digital health information and services | 4.73 |
| 6 | Science communication on research with health data and the transfer of scientific findings into practice | 5.25 |
| 7 | Automation of communication processes and collaboration between healthcare stakeholders | 5.26 |
| 8 | Science communication via digital and social media | 5.47 |
| 9 | Evaluation of the communication quality of digital applications (e.g., chatbots) between healthcare service providers (e.g., doctors and patients) | 6.74 |
