# Supplementary material for: Building consensus on core teaching content of a digital public health curriculum: a Delphi study with public health experts in Germany
Source: Front Public Health. 2026 Jun 19;14:1799393. doi: 10.3389/fpubh.2026.1799393 (PMC13328418; doi:10.3389/fpubh.2026.1799393)
Supplement: Supplementary file 3 [file Table_3.docx]

# Supplementary Material 3: Alignment of teaching content with established competence frameworks

## Table A14: Alignment of teaching content with the WHO Global competency and outcomes framework for the essential public health functions (2024)

| **WHO**  **competencies** | **Reflected in our list of included teaching contents** | **Final decision for teaching content** |
| --- | --- | --- |
| Promotes health equity among individuals and communities | Design of culturally appropriate and layperson-friendly digital health information and services | Included |
|  | Methods of diversity-sensitive data collection | Included |
|  | The relationship between the use of digital innovations and (the reduction or increase in) health and social inequality | Included |
|  | Definition, understanding, analysis, and promotion of digital and information technology literacy | Included |
|  | Acceptance and willingness to use digital health services among the population (distribution by population groups and influencing factors) | Included |
|  | User-friendliness of digital health services across all age groups (universal design and human-computer interaction) | Included |
| Enables people to increase control over, and to improve, their health and lives | Not discussed as a specific digital public health-related teaching content. |  |
| Fosters inclusive and participatory approaches to public health that embrace cultural diversity and inclusion | Participatory approaches and user orientation for digital applications (e.g., acceptance research) | Included |
| Takes an evidence-informed approach to decision-making | Use of health data and information technologies for policy makers (data-informed policy) | Included |
| Applies systems thinking to public health problem-solving | Not discussed as a specific digital public health-related teaching content. |  |
| Adapts to unexpected or rapidly changing situations | Not discussed as a specific digital public health-related teaching content. |  |
| Communicates actively and attentively | Health communication via digital and social media | Included |
|  | Science communication via digital and social media | Included |
| Conveys information purposefully, including through trusted platforms and key partners | Use of digital communication channels (computer-based, web-based, multimedia processes) | Included |
|  | Automation of communication processes and collaboration between healthcare stakeholders | Included |
| Adapts communication to the contextual goals, needs, urgency, and sensitivity of the situation | Science communication on research with health data and the transfer of scientific findings into practice | Included |
| Engages in collaborative practice within and between defined teams | Digital applications for promoting interprofessional healthcare as part of innovative, interdisciplinary care concepts | Included |
| Engages in collaborative practice within partnerships and coalitions | Opportunities offered by digital networking, care provision, and uniformly available data for interprofessional healthcare | Included |
| Learns from, with, and about others | Not discussed as a specific digital public health-related teaching content. |  |
| Constructively manages tensions, conflicts, resistance, and opposition | Not discussed as a specific digital public health-related teaching content. |  |
| **WHO**  **competencies** | **Reflected in our list of included teaching contents** | **Final decision for teaching content** |
| Assesses data, information, and evidence from a range of sources | Strategies for tracking and containing pathogens and infectious diseases using health data | Included |
|  | Infodemiology (e.g., text analysis and natural language processing, search engine analysis, web scraping and data mining, or social media analytics) | Included |
|  | Methods for collecting and evaluating health data | Included |
|  | Use of new sources for health data (e.g., social media or wearables) | Included |
|  | Organization, management, and control of health data (FAIR principles) | Included |
| Promotes evidence-informed public health practice | (Development of) evidence-based digital health information | Included |
| Contributes to continuous quality improvement | Evaluation of the communication quality of digital applications (e.g., chatbots) between healthcare providers (e.g., physicians) and patients | Included |
|  | Quality criteria and standards for health technologies | Included |
| Works within the limits of competence and role responsibilities | Not discussed as a specific digital public health-related teaching content. |  |
| Demonstrates high standards of ethical conduct | Ethical and legal requirements for human-technology innovations | Included |
|  | Unethical behavior and social responsibility in the use of digital applications (in relation to vulnerable groups) | Included |
|  | General guarantee of security and confidentiality of health data | Included |
|  | Ethical use and implications of health data and management (e.g., consequences of data analysis and interpretation) | Included |
|  | Data protection laws and national laws on health data (e.g., the significance of the GDPR for Germany) | Included |
|  | Bioethical issues of digitalization (e.g., the use of Artificial Intelligence, Big Data, and algorithms in healthcare, patient sovereignty in telemedicine, nudging and behavioral manipulation, etc.) | Included |
| Engages in lifelong learning | Not discussed as a specific digital public health-related teaching content. |  |
| Adopts strategies to manage one’s own health and well-being | Implementation of health management processes using digital services and applications: promoting acceptance, user-friendliness, and change management | Included |
|  | Integration of digital services and applications into existing work processes | Included |

## Table A15: Alignment of teaching content with the ASPHER Core Curriculum Program for Public Health. Supporting Core Competencies for Public Health Professionals, Chapter 29 Digital Transformation in Public Health

| **ASPHER**  **competencies** | **Reflected in our list of included teaching contents** | **Final decision for teaching content** |
| --- | --- | --- |
| Basic IT and information  literacy | Definition, understanding, analysis, and promotion of digital and information technology literacy | Included |
| Digital communication | Use of digital communication channels (computer-based, web-based, multimedia processes) | Included |
| Digital health competency and literacy | Definition, understanding, analysis, and promotion of digital health literacy | Included |
| Emerging disruptive  technologies (AI) | Digital applications for promoting interprofessional healthcare as part of innovative, interdisciplinary care concepts | Included |
|  | Artificial Intelligence and Big Data in healthcare applications | Included |
| Digital health tools (hardware and software) | Handling of appropriate hardware and software for processing health information | Included |
|  | Hardware and software for processing information in healthcare and healthcare facilities | Excluded |
| Health informational systems knowledge and practice | Health information systems | Included |
| Patient-centered applications and education – no jargon; with photos | Design of culturally appropriate and layperson-friendly digital health information and services | Included |
| Services and information are easier to access (closer to people) | Technical barriers to access for patients (e.g., people with limited physical/mental abilities)  Access strategies for hard-to-reach target groups in digital health research | Included |
|  | Creating access to healthcare services and information through digitalization | Included |
|  | Digital target group-specific measures and access strategies for health promotion and prevention (with a particular focus on vulnerable groups) | Included |
| Usability across the age range | Design guidelines, phase, and process models of usability engineering for digital health applications (a process that runs parallel to traditional planning and development work to ensure the subsequent usability of a system) | Excluded |
|  | User-friendliness of digital health services across all age groups (universal design and human-computer interaction) | Included |
| Providing teaching and training (medical education) | Virtual and augmented reality in medical and public health education, training, and continuing education | Excluded |
| Understand and carry out  appropriate Boolean term search methodologies | Understanding and executing appropriate Boolean search methods | Included |
| Clinical decision-making support | Digital clinical decision support tools | Included |
| Collate, evaluate, and  analyze health data | Methods for collecting and evaluating health data | Included |
| Emerging sources of health data (social media,  wearables) | Use of new sources for health data (e.g., social media or wearables) | Included |
| Infodemic management and infodemiology | Infodemiology (e.g., text analysis and natural language processing, search engine analysis, web scraping and data mining, or social media analytics) | Included |

| **ASPHER**  **competencies** | **Reflected in our list of included teaching contents** | **Final decision for teaching content** |
| --- | --- | --- |
| Electronic medical records and distributed ledger  technologies | Blockchain and distributed ledger technologies in electronic patient records | Excluded |
| Wearables and sensors (for health prediction and health promotion) | Wearables and sensors for health prediction and health promotion | Included |
| Precision public health | Personalization and precision of digital public health services (e.g., digital twins) | Excluded |
| Basic medical informatics | Definitions, history, and introduction to health informatics | Excluded |
|  | Definitions, history, and introduction to medical informatics | Excluded |
|  | Definitions, history, and introduction to medical technology | Excluded |
| Data analysis (access to data, usability, biostatistics) | Use of software for computer-assisted evaluation of qualitative data and text analysis (e.g., MAXQDA and F4) | Excluded |
|  | Identification, evaluation, and use of secondary data (e.g., Google searches, biostatistics, etc.) | Excluded |
| GPS and proximity data in pandemics | Use of GPS and proximity data (e.g., in pandemics) | Excluded |
| Pathology/X-ray software | Pathology and X-ray software | Excluded |
| Computerized Care Plans | Software-supported care documentation (e.g., via telecare, DiGA, DiPA, care robotics, chatbots, or computerized care plans) | Excluded |
| Infectious disease tracking, tracing, and containing | Strategies for tracking and containing pathogens and infectious diseases using health data | Included |
| Medication calculators | Medication calculators | Excluded |
| Genomics | Genomics (applications and self-testing mechanisms) | Excluded |
| Statistical software - Microsoft Excel, STATA, SPSS, SAS, R | Proficiency in statistical software (e.g., STATA, SPSS, SAS, R) | Included |
| Epidemiology data entry programs - Microsoft ACCESS, EpiData | Use of data entry programs (e.g., Microsoft ACCESS, EpiData) | Excluded |
| Epidemiological calculators - EpiTab, OpenEpi, WinPEPI, PS (Power and Sample Size) | Use of software for calculating epidemiological study cohorts (e.g., Plug and Chug Epi Calculator, EpiTab, OpenEpi, WinPEPI, PS, etc.) | Excluded |
| Data Transfer Software - StatTransfer | Use of data transfer software (e.g., StatTransfer) | Excluded |
| Security, privacy, and  confidentiality | General guarantee of security and confidentiality of health data | Included |
| GDPR (Data protection laws) and national laws on health data privacy and  security | Data protection laws and national laws on health data (e.g., the significance of the GDPR for Germany) | Included |
| Organize, manage, and  govern health data (FAIR principles) | Organization, management, and control of health data (FAIR principles) | Included |
| Structural interoperability | Structural interoperability (exchange of data in the correct format and structure between systems and organizations via standardized data formats and communication protocols) | Included |
| Organisational  interoperability | Organizational interoperability (effective collaboration and communication between different organizations within the system despite differing structures and processes) | Included |

| **ASPHER**  **competencies** | **Reflected in our list of included teaching contents** | **Final decision for teaching content** |
| --- | --- | --- |
| Semantic interoperability | Semantic interoperability (application of standardized terminologies, classification systems, and data formats to ensure that exchanged data is understood and interpreted in the same way by the systems and organizations involved) | Included |
| Investment in technical  infrastructure | Overview of investments in the technical infrastructure of the healthcare system | Excluded |
| WHO resources for digital transformation | WHO sources and resources for the digital transformation of healthcare systems | Excluded |
| Bioethics | Bioethical issues of digitalization (e.g., the use of Artificial Intelligence, Big Data, and algorithms in healthcare, patient sovereignty in telemedicine, nudging and behavioral manipulation, etc.) | Included |
| Ethical, legal, and regulatory requirements | Ethical and legal requirements for human-technology innovations | Included |
|  | Application and use of ELSI issues (ethical, social, and legal issues in health technology assessments) | Included |
| Data and digital equity | Methods of diversity-sensitive data collection | Included |
|  | The relationship between the use of digital innovations and (the reduction or increase in) health and social inequality | Included |
|  | Acceptance and willingness to use digital health services among the population (distribution by population groups and influencing factors) | Included |
| System readiness for an emergency | Strengthening the resilience and responsiveness of the healthcare system in emergency situations through digital strategies and applications (e.g., early warning systems, AI-supported triage tools, training, etc.) | Included |
| Risk management and risk communication | Risk management and risk communication in healthcare (e.g., cybersecurity, backup solutions from healthcare providers, and data leaks) | Excluded |
| Risk and its influence | Information about the risks and opportunities of digitization in health and disease | Included |
| Data ethically used to shape service provision | Ethical use and implications of health data and management (e.g., consequences of data analysis and interpretation) | Included |
|  | Unethical behavior and social responsibility in the use of digital applications (in relation to vulnerable groups) | Included |
| Certification of public health services | Certification of digital public health services | Excluded |
| Robotics | Robotics (medicine, surgery, and pharmacy) | Excluded |
| Use of drones for population surveillance | Drones and delivery of healthcare services (medications) | Excluded |
|  | Digital surveillance (including health protection against infectious and non-infectious diseases) | Included |
| Social media | Media strategies for health promotion (e.g., social media) | Included |
| Spread of information across digital networks | Opportunities offered by digital networking, care provision, and uniformly available data for interprofessional healthcare | Included |
|  | (Digital) networks in healthcare: Connecting features of digitalization (freedom from space and time limits) | Excluded |
| Communication strategies | Health communication via digital and social media  Science communication via digital and social media | Included |

| **ASPHER**  **competencies** | **Reflected in our list of included teaching contents** | **Final decision for teaching content** |
| --- | --- | --- |
| Health misinformation | Health-related misinformation (misinformation, “fake news”) | Included |
| Big technology’s role in  public health | The role of big tech companies in public health | Excluded |
| Telecommunications  (patients/population  and health service) | Telecommunications, fundamentals of network technologies (e.g., the Internet), and principles of data transmission/security | Excluded |
| Food safety alerts | Digital risk communication on food safety | Excluded |
| Digital collaboration and  relevant stakeholder  involvement | Automation of communication processes and collaboration between healthcare stakeholders | Included |
| Futures 2030 commission | Futures 2030 Commission (purpose, goals, milestones, etc.) | Excluded |
| Multimethod systems  (referencing, plagiarism, and grammatical system),  Grammarly | Use of multi-method systems (referencing, plagiarism, and grammar systems) | Excluded |
| Bibliographic and referencing systems - EndNote, Reference Manager, RefWorks, and ProCite | Working with bibliographic and referencing systems (e.g., EndNote, Reference Manager, RefWorks, and ProCite) | Included |
| Environmental health | Modeling methods for epidemics of infectious and non-infectious diseases and environmental changes | Included |
| Research methodologies | Quantitative and qualitative methods for digital health topics | Included |
|  | Procedural models for developing frameworks for (complex) digital interventions | Included |
|  | Assessment of health technologies (assessment of technological consequences) | Included |
|  | Dealing with AI-based applications in public health research | Included |
|  | Quality criteria for digital empirical health research | Included |
|  | Participatory approaches and user orientation for digital applications (e.g., acceptance research) | Included |
| Research use for  policymakers | Use of health data and information technologies for policy makers (data-informed policy) | Included |
| Social health experience | Definition and introduction to the digital divide: forms and models of the digital divide | Included |
