# Supplementary material for: Building consensus on core teaching content of a digital public health curriculum: a Delphi study with public health experts in Germany
Source: Front Public Health. 2026 Jun 19;14:1799393. doi: 10.3389/fpubh.2026.1799393 (PMC13328418; doi:10.3389/fpubh.2026.1799393)
Supplement: Supplementary file 4 [file Table_4.docx]

## Supplementary Material 3: Delphi studies in social and health sciences – recommendations for an interdisciplinary standardized reporting (DELPHISTAR)

| **Topic** | **Section** | **Item** | **Checklist Item** | **Addressed in section…** |
| --- | --- | --- | --- | --- |
| **I**  **Title and Abstract** |  | 1 | Identification as a Delphi procedure in the title | yes |
|  |  | 2 | Identification as a Delphi procedure in the abstract | yes |
|  |  | 3 | Structured abstract | yes |
| **II**  **Context** | **Formal** | 4 | Information about the sources of funding | Funding |
|  |  | 5 | Information about the team of authors and/or researchers (e.g., discipline, institution) | Materials and methods |
|  |  | 6 | Information about the method consulting | Questionnaire design and study procedure |
|  |  | 7 | Information about the project background | Introduction |
|  |  | 8 | Information about the study protocol | NA |
|  | **Content** | 9 | Justification of the chosen method (Delphi procedure) to answer the research question | Study design |
|  |  | 10 | Aim of the Delphi procedure (e.g., consensus, forecasting) | Introduction |
| **III**  **Method** | **Body and**  **integration of knowledge** | 11 | Identification and elucidation of relevant expertise, spheres of experience, and perspectives (e.g., theory, practice, affected groups, disciplines) | Expert selection |
|  |  | 12 | Handling of knowledge, expertise, and perspectives that are missing or have been deliberately not integrated | NA |
|  |  | 13 | Basic definition of expert^1^ | Expert selection |
|  | **Delphi**  **variations** | 14 | Identification of the type of Delphi procedure and potential modifications (e.g., classic Delphi, real-time Delphi, group Delphi) | Study design |
|  |  | 15 | Justification of the Delphi variation and modifications, including during the Delphi process, if applicable | Questionnaire design and study procedure |
|  | **Sample of**  **experts** | 16 | Selection criteria for the experts (per round, if there are different expert groups) | Expert selection |
|  |  | 17 | Identification of the experts | Expert selection |
|  |  | 18 | Information about recruiting and any subsequent recruiting of experts | Expert selection |
|  | **Survey** | 19 | Elucidation of the content development for the questionnaire^2^ | Questionnaire design and study procedure |
|  |  | 20 | Description of the questionnaire (content and structure) | Questionnaire design and study procedure |
|  | **Delphi rounds** | 21 | Number of Delphi rounds | Questionnaire design and study procedure |
|  |  | 22 | Information about the aims of the individual Delphi rounds | Questionnaire design and study procedure |

| **Topic** | **Section** | **Item** | **Checklist Item** | **Addressed in section…** |
| --- | --- | --- | --- | --- |
| **III**  **Method** | **Delphi rounds** | 23 | Disclosure and justification of the criterion for discontinuation | Questionnaire design and study procedure |
|  | **Feedback** | 24 | Information about what data was reported back per round | Questionnaire design and study procedure |
|  |  | 25 | Information on how the results of the previous Delphi round were fed back to the experts surveyed (e.g., via frequencies, mean values, measures of dispersion, listing of comments) | Questionnaire design and study procedure; Data analysis |
|  |  | 26 | Information on whether feedback was differentiated by specific groups (e.g., by field of expertise, institutional affiliation) | Questionnaire design and study procedure |
|  |  | 27 | Information about how dissent and unclear results were handled | Questionnaire design and study procedure |
|  | **Data**  **analysis** | 28 | Disclosure of the quantitative and qualitative analytical strategy | Data analysis |
|  |  | 29 | Definition and measurement of consensus | Questionnaire design and study procedure |
|  |  | 30 | Information on group-specific analysis or weighting of experts (e.g., theory vs. practice, discipline-specific analysis) | NA |
| **IV**  **Results** | **Delphi**  **process** | 31 | Illustration of the Delphi process (e.g., in a flow chart) | Figure 1 |
|  |  | 32 | Information about special aspects during the Delphi process (e.g., deviations from the intended approach with justification) | Questionnaire design and study procedure |
|  |  | 33 | Number of experts per round (both invited and participating) | Figure 1 |
|  | **Results** | 34 | Presentation of the results for each Delphi round and the final results | Core teaching content in digital public health; Most important teaching content; Alignment of teaching contents with established competence frameworks |
| **V**  **Discussion** | **Quality of findings** | 35 | Highlighting the findings from the Delphi study | Discussion of results |
|  |  | 36 | Validity of the results (e.g., transferability of the findings) | Discussion of results |
|  |  | 37 | Reliability of the results (e.g., split-half, inter-rater reliability) | Discussion of results |
|  |  | 38 | Reflection on potential limitations (e.g., distortion, skewing, bias) | Strengths and limitations |

^1^ “Experts” are the participants; this can be people from academia, practice, or representatives of lived experience (e.g., patients, family members).

^2^ The term “questionnaire” stands for the survey instrument regardless of whether quantitative or qualitative items are integrated or weighted.
